# Forest Degradation as a Potential Driver of Shifting Baseline Syndrome in Northeastern Brazil: A Case Study

**DOI:** 10.1007/s00267-026-02456-7

**Published:** 2026-04-16

**Authors:** Wyllamys Fernandes da Silva, Diego Centeno-Alvarado, Taline Cristina da Silva, Marcelo Alves Ramos

**Affiliations:** 1 Programa de Pós-Graduação em Etnobiologia e Conservação da Natureza, Universidade Federal Rural de Pernambuco, Recife, Pernambuco Brazil; 2 Programa de Pós-Graduação em Biologia Vegetal, Universidade Federal de Pernambuco, Recife, Pernambuco Brazil; 3 Laboratório de Etnobiologia e Conservação de Ecossistemas, Colegiado de Biologia, Universidade Estadual de Alagoas, Palmeira dos Índios, Alagoas Brazil; 4 Laboratório de Estudos Etnobiológicos, Universidade de Pernambuco, Campus Mata Norte, Nazaré da Mata, Pernambuco Brazil

**Keywords:** Change blindness, Conservation, Generational amnesia, Local ecological knowledge, Shifting baseline syndrome

## Abstract

Human-induced environmental changes cause shifting baseline syndrome (SBS), where degraded conditions redefine ‘normal’, leading to generational differences in perception and ultimately influencing biodiversity conservation strategies. In this case study, we examine (1) whether forest degradation influences SBS through local ecological knowledge (LEK) of woody medicinal plants, and (2) whether the cultural transmission of LEK contributes to resilience against SBS, focusing on two communities in Pernambuco state, Brazil, with distinct characteristics: *Sítio Cutia*, a frequently used forested area with high degradation, and *Sítio Limeirinha*, a monitored forested area with lower degradation. We conducted semi-structured interviews with residents (18+) grouped by age groups. To assess the effects of forest degradation on SBS, we analyzed age group differences in knowledge of woody medicinal plant richness (using GLMs) and perceptions of plant availability (using PERMANOVA and nMDS). LEK transmission networks were examined to evaluate whether cultural transmission contributes to resilience against SBS (using interaction degree distribution under different extinction scenarios). Contrary to expectations, our study suggests forest degradation does not necessarily intensify SBS, as perceptions of ecological change remained relatively stable across age groups. Within the monitored forest site, LEK transmission networks showed higher potential for resilience, indicating that intact ecological processes can help maintain accurate environmental knowledge over time. While the findings are specific to the socio-ecological systems examined, they highlight the value of integrating biodiversity conservation with cultural knowledge transmission. Efforts to mitigate SBS should integrate ecosystem conservation and age-structured LEK maintenance.

## Introduction

Since the industrial era, human activities have significantly altered natural ecosystems, leading to habitat loss and biodiversity decline (Tilman and Lehman 2001; Ellis et al. 2010; Adla et al. 2022). These changes may influence public perception of the natural environment, often distorting or diminishing awareness of the severity of ongoing environmental transformations (Soga and Gaston 2018). Shifting baseline syndrome (hereafter referred to as SBS) describes the gradual normalization of altered environmental conditions, as individuals base their perception of what is “natural” on current, often degraded (i.e., a condition of anthropogenic-induced stunted ecological succession, where the processes driving forest dynamics are weakened or significantly restricted; Ghazoul et al. 2015), ecosystem states without considering historical reference points (Pauly 1995; Soga and Gaston 2018). This shift obscures the full extent of human impacts on nature, allowing degradation to redefine what is considered acceptable (Soga and Gaston 2018). Environmental perceptions evolve across different ages, with younger individuals often adopting lower baseline standards for defining a healthier environment, particularly in the context of climate change, forest degradation, resource depletion, and biodiversity loss (Pauly 1995; Papworth et al. 2009; Soga and Gaston 2018, 2021, 2024). The negative impacts of SBS on global conservation are substantial. One key issue is its effect on stakeholder interest, involvement, and support for conservation efforts, which can shift based on what is considered acceptable in increasingly degraded conditions (Papworth et al. 2009; Hayhow et al. 2019; Soga and Gaston 2018). Another consequence is the lowering of expectations for what constitutes a desirable environment, leading to reduced ambitions and less effort in restoration initiatives (Soga and Gaston 2018). Additionally, the use of inappropriate baselines may diminish public motivation to engage in conservation activities (Bilney 2014; Soga and Gaston 2018), such as mitigating forest degradation.

Despite the importance of SBS in lowering these standards, it has received limited attention, and its causes and consequences remain poorly understood (Soga and Gaston 2018). However, a growing body of evidence highlights the presence of SBS in environmental perceptions and local ecological knowledge (hereafter referred to as LEK) across diverse socio-environmental contexts. These include plant knowledge among indigenous foraging-horticulturalist societies in the Bolivian Amazon (Fernández-Llamazares et al. 2015); perceptions of change in game invertebrates among coastal fishing communities in central Baja California, Mexico (Sáenz-Arroyo et al. 2005) and in East Africa (Katikiro 2014), coral reef fishing communities in the Raja Ampat Archipelago, Indonesia (Ainsworth et al. 2008), and hunting communities in Central Africa, including Gabon and Equatorial Guinea (Papworth et al. 2009); perceptions of water resource quality among indigenous communities in the Alaskan Arctic (Alessa et al. 2008); and perceptions of climate change impacts among remote indigenous communities in the Alaskan Subarctic (Herman-Mercer et al. 2016). Across these systems, younger individuals often underestimate resource depletion and the magnitude of environmental change compared to older individuals. A major cause of SBS is the decreasing interaction between people and the natural environment, leading to a growing disconnect from nature (Soga and Gaston 2018). Younger individuals, especially children, across the world now spend considerably less time outdoors than in previous years, often substituting outdoor experiences with digital entertainment (Soga and Gaston 2016, 2018; Soga et al. 2018). At the same time, knowledge of natural history, including basic skills such as identifying plants, is steadily declining, especially in developed countries (Pilgrim et al. 2008; Tewksbury et al. 2014). This decline continues despite the rapid growth of accessible natural history information made possible by advances in technology (Tosa et al. 2021). However, there are ways to mitigate the loss of interactions and familiarity with nature, such as implementing protected areas or enforcing specific forest management restrictions as strategies to mitigate forest degradation (Velazco et al. 2022). In these areas, interactions with a diverse range of species and proximity to natural environments enhance communities’ understanding of environmental changes (Velazco et al. 2022; Iniesta-Arandia et al. 2015).

In addition, SBS can be exacerbated by generational amnesia, or the unperceived loss of knowledge across age groups (Jones et al. 2020; Spennemann 2022). When communication among different age groups weakens, the baseline for what is considered a ‘normal’ ecological condition gradually erodes, limiting awareness of the long-term ecological change (Jones et al. 2020). This breakdown in transmission, often driven by changes in lifestyle, migration, or reduced interaction with nature, undermines the maintenance of LEK and leads to the forgetting or overlooking of historical ecological information (Soga and Gaston 2016, 2018). Conversely, resilient knowledge transmission networks can help buffer against SBS. Even as environmental conditions shift, older individuals can pass on information about past states of the ecosystem, enabling younger individuals to recognize that changes have occurred (e.g., Jardine 2019). Furthermore, in less degraded areas, ecological integrity permits traditional practices to continue (Ruifei and Gavin 2016; Moloise et al. 2023), potentially creating natural settings for the sharing of knowledge between individuals from different age groups. Degraded environments, on the other hand, diminish the perceived value and opportunities of these practices (Ruifei and Gavin 2016; Moloise et al. 2023), which could weaken the transmission of knowledge and potentially exacerbate SBS. Strengthening and nurturing knowledge exchanges across age groups, particularly in natural contexts, can help counteract the weakening of knowledge transmission caused by degraded environments, thereby mitigating SBS (Soga and Gaston 2016, 2018).

These considerations motivated our case study into the phenomenon, with two main objectives: (1) to assess the influence of forest degradation status on SBS, using evidence from LEK, and (2) to evaluate the impact of forest degradation on the potential of cultural transmission of LEK to enhance resilience against SBS. We focused on woody medicinal plants as a model, given their strong connection to biocultural heritage, which is largely passed down through oral traditions (Davis and Choisy 2024). The study was conducted in the Atlantic Forest remnants of Pernambuco, northeastern Brazil, chosen for their status as one of the most degraded regions of the Atlantic Forest due to extensive deforestation and fragmentation in recent decades (Dias et al. 2023), making them a relevant context for exploring the effects of environmental change on LEK. To investigate this, we tested the hypotheses that the degradation status of forested areas influences: (1) knowledge of diversity and perceptions of the availability of woody medicinal plants across different age groups, through changes in direct experience and familiarity; and (2) the potential contribution of cultural transmission of LEK to resilience against SBS, through changes in opportunities for knowledge exchange across age groups. We predict that: (1) differences in knowledge of plant diversity and perceptions of availability of woody medicinal plants between younger and older individuals will be greater in areas adjacent to degraded forests than in monitored forests, with older age groups expected to report higher diversity and more pronounced changes in availability (i.e., emergence of SBS); and (2) in areas adjacent to degraded forests, the cultural transmission network of LEK, particularly regarding medicinal plant use, will be disrupted, potentially reducing resilience against SBS.

## Material and Methods

### Study Site: Communities Studied

**Fig. 1:**
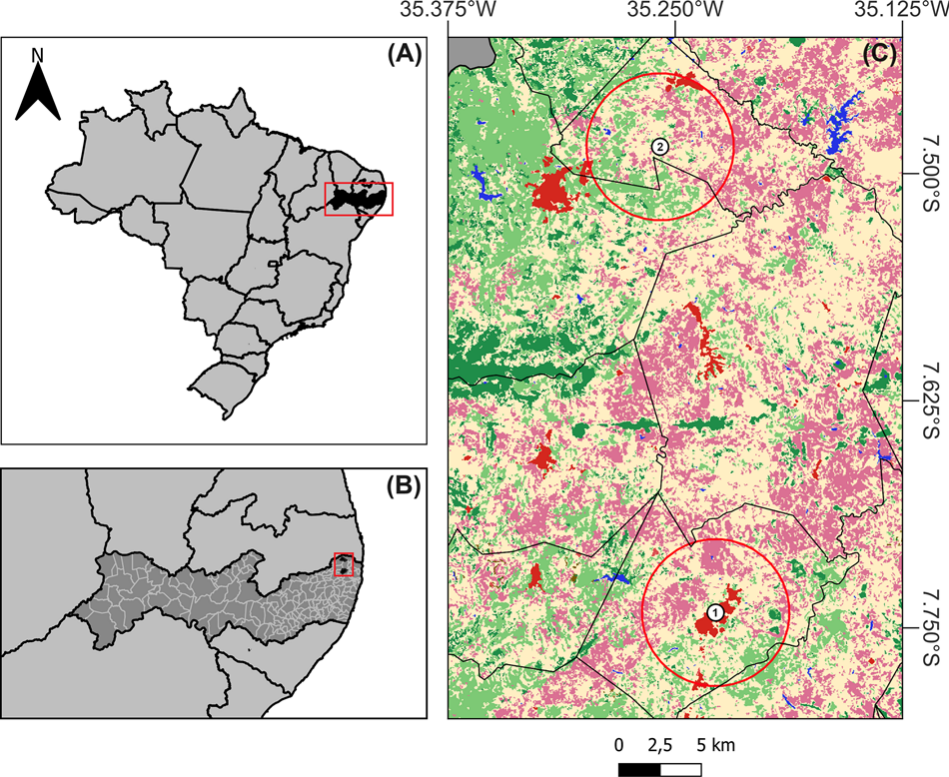
Map of Pernambuco, Brazil, shown in black (**A**), and the municipalities of Ferreiros and Nazaré da Mata, also highlighted in black (**B**), along with the two studied communities (**C**) (1: *Sítio Cutia*, Ferreiros, degraded forest area; 2: *Sítio Limeirinha*, Nazaré da Mata, monitored forest area). Green-shaded areas indicate regions with greater natural forest cover, while red and purple-shaded areas represent anthropized regions (e.g., sugarcane plantations). Red circles represent buffers with a 2.5 km radius, used only to visually illustrate the approximate spatial extent of each community in relation to forest degradation

Our study was conducted in two communities in Pernambuco state, northeastern Brazil: *Sítio Cutia*, located in the municipality of Ferreiros (7°29’06.9” S, 35°15’29.9” W), and *Sítio Limeirinha*, in the municipality of Nazaré da Mata (7°44’30.0” S, 35°13’40.0” W) (Fig. 1). These sites are situated in the humid coastal regions dominated by the wet tropical forest, commonly referred to as the Atlantic Forest. This area is regarded as one of the most fragmented sections of the Atlantic Forest, where sugarcane plantations are the predominant land use (Ranta et al. 1998; Siqueira Filho et al. 2007).

#### Sítio Cutia

*Sítio Cutia* (also referred to as a *degraded forest area*; municipality of Ferreiros) (Fig. 1C) is adjacent to a commonly used forested area that is considered degraded due to fires, selective logging, and wood fuel collection, primarily driven by the expansion of sugarcane monoculture (*Saccharum officinarum* L.) (Araújo et al. 2024). The community comprises 170 inhabitants, including 133 adults, residing in 58 households (pers. comm.). Public services are absent, and the primary economic activity in the area is agriculture, particularly sugarcane cultivation carried out on nearby large farms, alongside subsistence farming of yam (*Dioscorea cayennensis* Lam.), cowpea (*Vigna unguiculata* (L.) Walp.), sweet potato (*Ipomoea batatas* (L.) Lam.), cassava (*Manihot esculenta* Crantz), lettuce (*Lactuca sativa* L.), and coriander (*Coriandrum sativum* L.), among other crops grown for self-consumption and small-scale commercialization (Araújo et al. 2024; pers. comm.). Additionally, the local population consists of retirees receiving benefits from the National Social Security Institute, workers employed in nearby large farms and sugar mills, and families supported by Bolsa Família (pers. comm.), a conditional cash transfer family welfare federal program (Soares 2011).

#### Sítio Limeirinha

*Sítio Limeirinha* (also referred to as a *monitored forest area*, i.e., a low degradation level; municipality of Nazaré da Mata) (Fig. 1C) is located near the *Limeirinha* sugarcane processing plant and adjacent to the *Mata de Alcaparra*, an 83.8-hectare preserved fragment of Atlantic Forest (pers. comm.). The *Mata de Alcaparra* is continuously monitored by a specialized team to prevent fires, wildlife capture, illegal hunting and fishing, and forest degradation. This monitoring is conducted by the *Limeirinha* sugarcane operation as part of their environmental surveillance program to protect the surrounding natural areas (pers. comm.). The community consists of 241 inhabitants, including 194 adults, residing in 87 households (pers. comm.). The local economy relies primarily on rural labor, particularly sugarcane harvesting, along with government assistance programs such as *Bolsa Família*. Community members are permitted to access the forest to collect dry woody material. Long-term residents report that, due to company-led protection efforts, the forest has experienced minimal modifications in recent decades (pers. comm.).

### Forest Degradation Level Assessment

Based on a combination of site-level data and landscape-scale indicators of forest loss, we divided the two case-study sites into two categories to describe the degree of forest degradation in the study area: (1) *degraded forest area* (*Sítio Cutia*) and (2) *monitored forest area* (*Sítio Limeirinha*). The state of the nearby forest area served as the basis for classification at the site level. On the one hand, small forest patches that are subject to frequent fires, selective logging, and significant influence from the nearby sugarcane monoculture are linked to the *degraded forest area* (*Sítio Cutia*). On the other hand, a preserved 83.8-ha forest fragment that is constantly monitored to prevent fires, illicit logging, and other degradation processes is adjacent to the *monitored forest area* (*Sítio Limeirinha*). At the landscape-scale, we characterized recent historical forest disturbance around each community using the Global Forest Change dataset (2000–2024; Hansen/UMD/Google/USGS/NASA; Hansen et al. 2013). Forest loss during 2000–2024 was extracted as per-pixel indicators from the ‘lossyear’ band (0 = no loss, 1–20 = loss detected in 2001–2024, respectively), not in physical units; values were averaged across grid points to obtain a single metric of historical forest loss intensity representing the surrounding landscape of each community. *The degraded forest area* (*Sítio Cutia*: 10.98 ± 8.01, mean ± standard deviation) had a higher metric than the *monitored forest area* (*Sítio Limeirinha*: 5.07 ± 4.38).

### Ethnobotanical Data Collection

Initial visits were conducted to gather information from local leaders and community health agents in both communities. All local residents aged 18 and over who agreed to participate were included in the study. Participants were grouped into seven age groups, defined by 10-year intervals, which provides a standardized and objective classification of age structure, following Fernández-Llamazares et al. (2015): (1) 18–27 years, (2) 28–37 years, (3) 38–47 years, (4) 48–57 years, (5) 58–67 years, (6) 68–77 years, (7) ≥78 years. Data collection occurred in three stages between May and July 2023. The first stage involved semi-structured interviews (Albuquerque et al. 2014) using the ‘free listing’ technique, where participants were asked to list medicinal plants known to them in the forest. Plants were categorized as woody or non-woody based on a pre-selected model and then classified as native or exotic. For exotic species, the date of their introduction was reviewed from available sources. Because most exotic plants were introduced more than a century ago, they likely have a long history of use spanning several age groups; therefore, for further analysis, we did not separate native and exotic species. Additionally, the ‘reading back’ technique was employed to allow participants to recall and add plants to their list (Albuquerque et al. 2014). Based on the information retrieved from the interviews, we determined the knowledge of the diversity (i.e., richness) of woody medicinal plants for each participant. The second stage, conducted in follow-up home visits, focused on participants’ perceptions of the availability of woody medicinal plants, using their childhood or first contact with the resource as a reference. Participants rated the ten most frequently cited native woody plants among all individuals, aided by visual resources such as tree figures. They were asked whether they perceived an increase (1), decrease (-1), or no change (0) in plant availability compared to their childhood or first contact. These ratings were recorded for each of the ten plants and subsequently organized into a matrix. The third stage involved assessing the cultural transmission of medicinal plant knowledge. Participants were asked whether they had learned about the medicinal uses of woody plants (e.g., angico, *Anadenanthera colubrina* var. cebil (Griseb.) Altschul, for treating ‘rheumatism’) from others. They were also asked whether they had taught others about these uses. Participants could indicate from whom they had learned or to whom they had taught, specifying relationships (e.g., parent, elder, other community member), although the type of source was not formally analyzed.

At *Sítio Cutia*, a total of 88 participants (48 women, 40 men) were involved in the first stage, with 85 participants (46 women, 39 men) in the second stage. In the third stage, which evaluated the cultural transmission of knowledge, the learning vector included 71 participants (40 women, 31 men), while the teaching vector included 56 participants (34 women, 22 men). The age of women ranged from 20 to 84 years, and the age of men from 19 to 85 years. The length of residence in the community varied from 2 to 85 years [41.1 ± 20.7 (mean ± SD) years]. At *Sítio Limeirinha*, 130 participants (68 women, 62 men) took part in the first stage, 125 participants (68 women, 54 men) in the second stage, and the third stage included 94 participants in the learning vector (47 women and 47 men) and 97 participants in the teaching vector (54 women and 43 men). The age of women ranged from 18 to 91 years, and the age of men from 18 to 86 years. The length of residence in the community varied from 1.5 to 71 years (35.1 ± 17.7 years). Not all potential participants were interviewed due to refusals or health-related issues.

### Statistical Analyses

#### Assessment of Forest Degradation Impact on Shifting Baseline Syndrome (SBS)

To assess how forest degradation impacts SBS, we first investigated how knowledge of the diversity (i.e., richness) of woody medicinal plants differs among participants from different age groups in communities adjacent to a degraded forest area (i.e., *Sítio Cutia*, municipality of Ferreiros) and a monitored forest area (i.e., *Sítio Limeirinha*, municipality of Nazaré da Mata). To evaluate this, we used generalized linear models (GLMs) with a Poisson error distribution, including both age group and forest degradation status as predictors, along with their interaction term. Species richness of woody medicinal plants known by each participant was used as the response variable. A model selection approach based on Akaike’s information criterion with small-sample correction (AICc) (Burnham and Anderson 2002) was applied to identify the best-supported models. When the best-supported model included significant effects of age group, differences among age groups were assessed within each site using Dunn’s multiple comparisons test. This approach allows direct testing of both site effects and age × site interactions, aligning the statistical analysis with the objective of the study.

Pairwise differences among age groups were assessed using Dunn’s multiple comparisons test.

Second, to analyze differences in the perception of the availability of woody medicinal plants across age groups, we conducted a permutational multivariate analysis of variance (PERMANOVA) and a non-metric multidimensional scaling (nMDS) analysis separately for each area. PERMANOVA compares beta diversity, reflecting differences in species availability among samples, while nMDS preserves dissimilarity relationships among samples, facilitating the visual interpretation of compositional variations (Anderson 2017). In both PERMANOVA and nMDS, changes in the perception of the availability of woody medicinal plants (i.e., using the perception matrix previously recorded from the interviews) were used as response variables, with age group as the predictor variable. Furthermore, we conducted nMDS analyses using Euclidean distances to visually represent variations in changes in the perception of woody medicinal plant availability among age groups.

#### Evaluation of the Impact of Forest Degradation on the Potential Contribution of Cultural Transmission of Local Ecological Knowledge (LEK) to Resilience Against SBS

To examine the impact of forest degradation on the potential contribution of the cultural transmission of LEK to resilience against SBS, we followed an approach based on constructing networks of teaching and learning regarding the medicinal uses of woody plants in both communities. First, we used a bipartite affiliation matrix, with age groups in the rows and woody medicinal plants in the columns. In the matrix, we recorded the total number of medicinal uses and woody plant species associated with the participants from each age group. For example, if a participant mentioned the use of angico, *A. colubrina* var. *cebil*, to treat both ‘rheumatism’ and ‘cough’, these would be considered two distinct medicinal uses (i.e., interactions). Based on this data, we constructed a total of four networks: for each community, one network represented the learning vector (i.e., acquired knowledge of woody medicinal plants) and another represented the teaching vector (i.e., transmission of this knowledge to others). The construction of these networks was based on direct participant responses about whether they had learned or taught specific medicinal uses and, if applicable, from/to whom. These “vectors” thus represent empirically reported teaching and learning interactions rather than inferred patterns of knowledge alone. We then analyzed the degree distribution at the higher level (i.e., age groups) for each network and both vectors (learning and teaching) to evaluate their susceptibility to different network extinction scenarios (Ávila-Thieme et al. 2023), using it as a proxy for potential resilience against SBS. To analyze the degree distribution, we assessed the cumulative distribution of connections for each species (i.e., node) (Estrada 2007), considering only the higher-level structure in this study—age groups. The aim was to investigate whether the susceptibility of the network’s higher level to the removal of the most interconnected species was linked to the distribution of their degrees (Estrada 2007). The degree distribution was estimated by fitting three distributions: exponential, power law, and truncated power law, with model selection based on the Akaike Information Criterion (AIC), where the models with the lowest AIC values were chosen. Networks that exhibit a degree distribution following a power law are highly susceptible to the removal of the most connected nodes, while networks with an exponential degree distribution show the opposite (Albert and Barabási 2002; Dunne et al. 2002; Estrada 2007). In addition, truncated power-law distributions exhibit power-law behavior at lower node degrees but transition into an exponential decay at higher degrees (Mossa et al. 2002). This truncation makes the network less fragile than a pure power-law network but more vulnerable than an exponential one (Mossa et al. 2002). In cases where the network of a community is more susceptible to the removal of the most connected nodes, there is a higher probability of disrupting the cultural transmission of LEK and, consequently, lower resilience against SBS.

The analyses were conducted using the ‘*stats*’ (v. 4.3.0; R Core Team 2022), ‘*vegan*’ (v. 2.6-4; Oksanen et al. 2022), ‘*bipartite*’ (v. 2.20; Dormann et al. 2024), and ‘*NetworkExtinction*’ (v. 1.0.3; Corcoran et al. 2023) packages in the R environment (v. 4.1.3; R Core Team 2022).

## Results

At *Sítio Cutia* (adjacent to a degraded forest area), participants cited 75 woody medicinal plant species (Table 1). The most frequently mentioned plants were aroeira (*Astronium urundeuva* (M.Allemão) Engl.) (96.59%), caju roxo (*Anacardium occidentale* L.) (79.55%), angico (*A. colubrina* var. *cebil*) (60.23%), barbatimão (*Stryphnodendron adstringens* (Mart.) Coville) (55.68%), jenipapo (*Genipa americana* L.) (54.55%), juá (*Ziziphus joazeiro* Mart.) (45.45%), jatobá (*Hymenaea courbaril* L.) (29.55%), quixaba (*Sideroxylon obtusifolium* (Roem. & Schult.) T.D.Penn.) (28.41%), goiaba (*Psidium guajava* L.) (25.00%), and pitanga (*Eugenia uniflora* L.) (25.00%) (Table 1). At *Sítio Limeirinha* (adjacent to a monitored forest area), participants cited 74 woody medicinal plant species. The most frequently mentioned species were aroeira (*A. urundeuva*) (99.17%), barbatimão (*S. adstringens*) (76.67%), jenipapo (*G. americana*) (72.50%), jatobá (*H. courbaril*) (67.50%), juá (*Z. joazeiro*) (62.50%), caju roxo (*A. occidentale*) (59.17%), mutamba (*Guazuma ulmifolia* Lam.) (53.33%), angico (*A. colubrina* var. *cebil*) (36.67%), macaíba (*Acrocomia intumescens* Drude) (18.33%), and mulungu (*Erythrina velutina* Willd.) (14.17%) (Table 1). All these species are native to the region.

**Table 1:** Woody Medicinal Species Known and Used by the Communities of *Sítio Cutia* (Municipality of Ferreiros) and *Sítio Limeirinha* (Municipality of Nazaré da Mata), State of Pernambuco, Northeastern Brazil

Family	Species	Common name	Biogeographic origin	Approximate introduction period	Citation frequency (%)	
					*Sítio Cutia*	*Sítio Limeirinha*
Anacardiaceae	*Anacardium occidentale* L.	Caju roxo	Native	-	79.55	59.17
Anacardiaceae	*Astronium urundeuva* (M.Allemão) Engl.	Aroeira	Native	-	96.59	99.17
Anacardiaceae	*Mangifera indica* L.	Manga	Exotic	1700s^a^	1.14	2.50
Anacardiaceae	*Spondias dulcis* Parkinson	Cajarana	Native	-	2.27	0.83
Anacardiaceae	*Spondias mombin* L.	Cajá	Native	-	1.14	0
Anacardiaceae	*Spondias purpurea* L.	Seriguela	Native	-	0	1.67
Arecaceae	*Acrocomia intumescens* Drude	Macaíba	Native	-	0	18.33
Arecaceae	*Cocos nucifera* L.	Coco	Exotic	1500s^b^	6.82	0
Arecaceae	*Elaeis guineensis* Jacq.	Coco dendê	Native	-	0	5.83
Arecaceae	*Syagrus* sp.	Coco catolé	Native	-	4.55	4.17
Bignoniaceae	*Tabebuia* sp.	Pau d’arco	Native	-	10.23	6.67
Bignoniaceae	*Tabebuia serratifolia* (Vahl) G.Nichols.	Pau d’arco amarelo	Native	-	2.27	0.83
Bignoniaceae	*Tabebuia impetiginosa* (Mart. ex DC.) Standl.	Pau d’arco roxo	Native	-	6.82	5.83
Bombacaceae	*Pseudobombax* sp.	Barriguda	Native	-	4.55	0
Cordiaceae	*Cordia trichotoma* (Vell.) Arráb. ex Steud.	Frei Jorge	Native	-	1.14	0.83
Capparaceae	*Crataeva tapia* L.	Trapiá	Native	-	1.14	0.83
Combretaceae	*Terminalia catappa* L.	Castanhola	Exotic	1500s^c^	1.14	0
Combretaceae	*Thiloa glaucocarpa* (Mart.) Eichler	Sipaúba	Native	-	0	3.33
Euphorbiaceae	*Croton blanchetianus* Baill.	Marmeleiro	Native	-	20.45	0.83
Euphorbiaceae	*Jatropha molissima* (Pohl) Baill.	Pinhão branco	Native	-	1.14	0
Fabaceae	*Anadenanthera colubrina* var. *cebil* (Griseb.) Altschul	Angico	Native	-	60.23	36.67
Fabaceae	*Bauhinia cheilantha* (Bong.) Steud.	Mororó	Native	-	5.68	0
Fabaceae	*Bowdichia virgilioides* Kunth	Sucupira	Native	-	1.14	1.67
Fabaceae	*Caesalpinia echinata* Lam.	Pau Brasil	Native	-	0	1.67
Fabaceae	*Erythrina velutina* Willd.	Mulungu	Native	-	15.91	14.17
Fabaceae	*Geoffroea spinosa* Jacq.	Marí	Native	-	1.14	0
Fabaceae	*Inga vera* Willd.	Ingá	Native	-	0	0.83
Fabaceae	*Hymenaea courbaril* L.	Jatobá	Native	-	29.55	67.50
Fabaceae	*Libidibia ferrea* (Mart. ex Tul.) L.P.Queiroz	Jucá	Native	-	26.14	10.83
Fabaceae	*Machaerium aculeatum* Raddi	Espinho de judeu	Native	-	2.27	0
Fabaceae	*Machaerium* sp.	Espinheiro	Native	-	7.95	13.33
Fabaceae	*Mimosa caesalpiniifolia* Benth.	Sabiá	Native	-	2.27	0.83
Fabaceae	*Piptadenia retusa* (Jacq.) P.G.Ribeiro, Seigler & Ebinger	Jurema	Native	-	0	1.67
Fabaceae	*Prosopis juliflora* (Sw.) DC.	Algaroba	Exotic	1942^d^	1.14	0
Fabaceae	*Senegalia tenuifolia* (L.) Britton & Rose	Calombi	Native	-	6.82	3.33
Fabaceae	*Stryphnodendron adstringens* (Mart.) Coville	Barbatimão	Native	-	55.68	76.67
Fabaceae	*Swartzia flaemingii* Raddi	Jacarandá	Native	*-*	0	0.83
Fabaceae	*Tamarindus indica* L.	Tamarindo	Exotic	1600s^e^	1.14	5.83
Lauraceae	*Persea americana* Mill.	Abacate	Native	-	1.14	0
Lecythidaceae	*Eschweilera ovata* (Cambess.) Mart. ex Miers	Imbiriba	Native	-	0	0.83
Lythraceae	*Punica granatum L*.	Romã	Exotic	1500s^f^	4.55	4.17
Malpighiaceae	*Malpighia emarginata* DC.	Acerola	Exotic	1956^g^	0	0.83
Malvaceae	*Guazuma ulmifolia* Lam.	Mutamba	Native	-	14.77	53.33
Meliaceae	*Azadirachta indica* A.Juss.	Nim	Exotic	1986^h^	0	0.83
Moraceae	*Artocarpus heterophyllus* Lam.	Jaca	Exotic	1600s^i^	1.14	0
Myrtaceae	*Eucalyptus* sp.	Eucalipto	Exotic		5.68	3.33
Myrtaceae	*Eugenia uniflora* L.	Pitanga	Native	-	25	5.83
Myrtaceae	*Plinia cauliflora* (Mart.) Kausel	Jabuticaba	Native	-	4.55	0
Myrtaceae	*Psidium guajava* L.	Goiaba	Native	-	25	13.33
Myrtaceae	*Psidium guineense* Sw.	Araçá	Native	-	1.14	0
Myrtaceae	*Syzygium cumini* (L.) Skeels	Azeitona	Exotic	1500s^j^	4.55	2.50
Nyctaginaceae	*Guapira noxia* (Netto) Lundell	João mole	Native	-	21.59	1.67
Rhamnaceae	*Ziziphus joazeiro* Mart.	Juá	Native	-	45.45	62.50
Rubiaceae	*Genipa americana* L.	Jenipapo	Native	-	54.55	72.50
Sapindaceae	*Cupania impressinervia* Acev. Rodr.	Cabatam	Native	-	0	1.67
Sapotaceae	*Sideroxylon obtusifolium* (Roem. & Schult.) T.D.Penn.	Quixaba	Native	-	28.41	1.67
Urticaceae	*Cecropia* sp.	Embaúba	Native	-	0	3.33
Vochysiaceae	*Callisthene fasciculata* Mart.	Campineiro	Native	-	12.50	0

Species are listed in alphabetical order of botanical families, along with local common names, biogeographic origin, and citation frequencies in each community

^a^Warschefsky and von Wettberg (2019)

^b^Gunn et al. (2011)

^c^Maria et al. (2021)

^d^Andrade et al. (2009)

^e^Yahia and Salih (2011)

^f^Associação dos Jovens Agricultores de Portugal (2017)

^g^Maciel et al. (2010)

^h^Moro et al. (2013)

^i^Freitas et al. (2017)

^j^Mussi (2018)

### Assessment of Forest Degradation Impact on Shifting Baseline Syndrome (SBS)

**Fig. 2:**
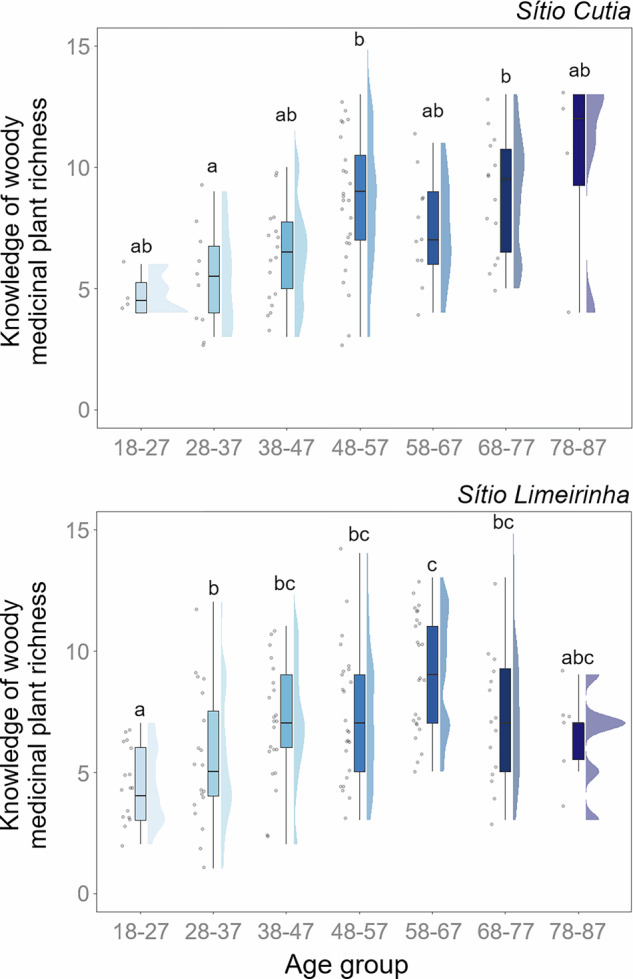
Significant effects of age group on knowledge of woody medicinal plant richness based on LEK in the communities of *Sítio Cutia* (Ferreiros; adjacent to a degraded forest area) and *Sítio Limeirinha* (Nazaré da Mata; adjacent to a monitored forest area), Pernambuco, Brazil. The figure displays median values of the number of species reported by each age group with interquartile ranges, maximum and minimum values represented by bars, raw data points, and half-cut bars illustrating data distribution. Statistically significant differences are indicated by different letters at the top of the bars

**Fig. 3:**
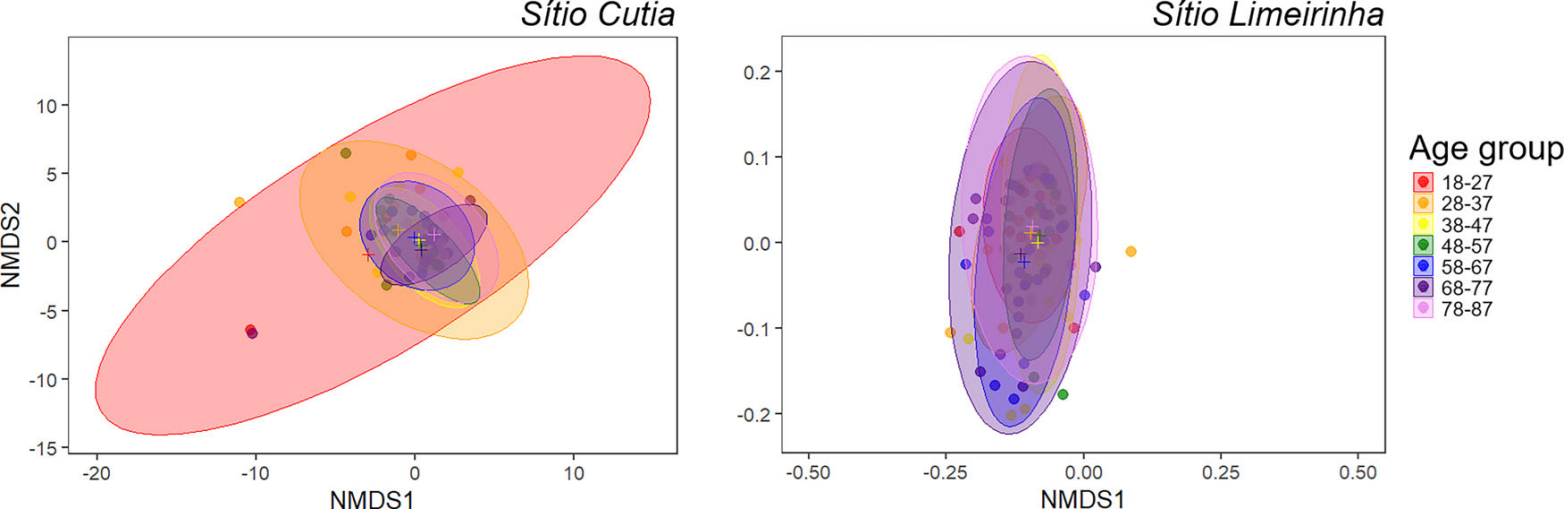
Non-metric multidimensional scaling (nMDS) plots for changes in the perception of woody medicinal plant availability. A single nMDS analysis was performed for each site to assess changes in perceived availability [2-D stress: *Sítio Cutia* (Ferreiros; adjacent to a degraded forest area): 0.13; *Sítio Limeirinha* (Nazaré da Mata; adjacent to a monitored forest area): 0.09]. Results are represented by seven distinct polygons based on age groups

The GLM tests revealed that age group was retained in the best-supported models, explaining knowledge of woody medicinal plant richness, while the interaction with forest degradation status was not supported based on AICc, suggesting that forest degradation did not modify age-related differences (Table 2). In *Sítio Cutia*, the community adjacent to the degraded forest area, Dunn’s multiple comparisons test indicated that knowledge of woody medicinal plant richness was significantly higher among participants aged 68–77 years (*p* = 0.04) and 48–57 years (*p* = 0.03) compared to those aged 28–37 years (Table S2 – Supplementary Material; Fig. 2). However, no significant differences were observed among other age groups. Conversely, in *Sítio Limeirinha*, the community adjacent to the monitored forest area, Dunn’s multiple comparisons test indicated that knowledge of woody medicinal plant richness was significantly higher among participants aged 38–47 (*p* = 0.01), 48–57 (*p* = 0.02), 58–67 (*p* = 0.001), and 68–77 (*p* = 0.02) years compared to those aged 18–27 years, as well as among individuals aged 58–67 years (*p* = 0.001) compared to those aged 28–37 years (Table S2 – Supplementary Material; Fig. 2). Moreover, no significant differences were observed among other age groups. Additionally, the perception of the availability of woody medicinal plants did not differ across age groups in either population [*Sítio Cutia* (adjacent to the degraded forest area): *p* = 0.34; *Sítio Limeirinha* (adjacent to the monitored forest area): *p* = 0.76] (Table S3 – Supplementary Material; Fig. 3).

**Table 2:** Summary of the Best-Supported Models Examining the Associations Between Woody Species Richness, Forest Degradation Status, and Age Groups in the Communities of *Sítio Cutia* (Municipality of Ferreiros) and *Sítio Limeirinha* (Municipality of Nazaré da Mata), State of Pernambuco, Northeastern Brazil

No.	Full model	Best-supported models	Explanatory variables retained	AICc	∆AICc	*R*^2^	Weight
*Woody species richness*							
1	*Richness* ~ Age group × Forest degradation status	1	(1) Age group	1072.7	0	0.22	0.56
		2	(1) Age group, (2) Forest degradation status	1074.7	1.01	0.23	0.34

### Evaluation of the Impact of Forest Degradation on the Potential Contribution of Cultural Transmission of Local Ecological Knowledge (LEK) to Resilience Against SBS

**Fig. 4:**
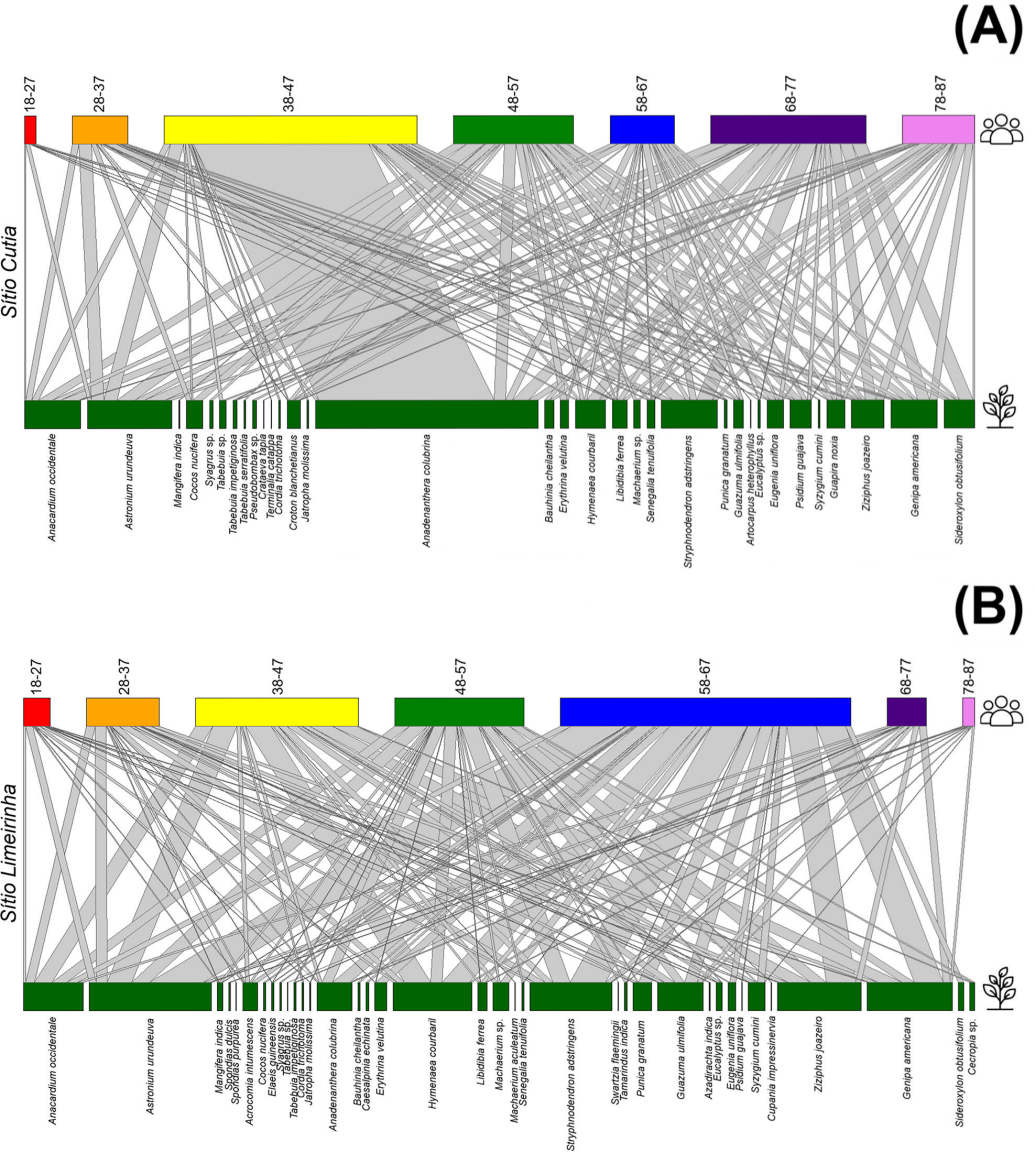
Cultural transmission of knowledge through learning vectors in the communities of *Sítio Cutia* (Ferreiros; adjacent to a degraded forest area) (**A**) and *Sítio Limeirinha* (Nazaré da Mata; adjacent to a monitored forest area) (**B**). The top section represents age groups (indicated by different colors), while the bottom section shows woody medicinal plant species (each represented by blue). Bars connect generations to plant species, illustrating knowledge transmission

**Fig. 5:**
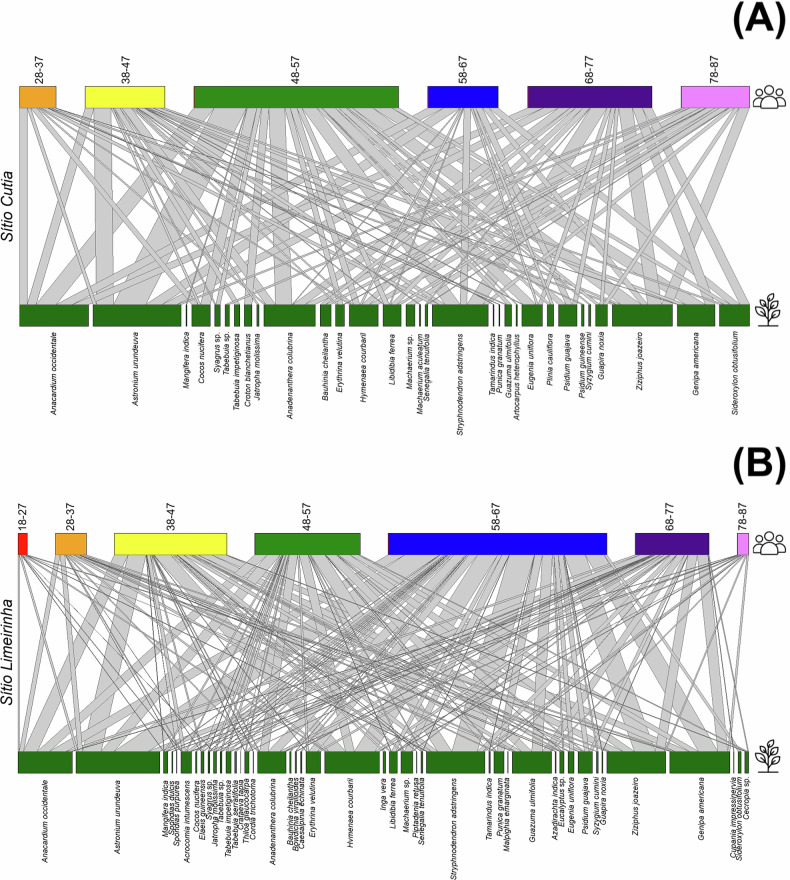
Cultural transmission of knowledge through teaching vectors in the communities of *Sítio Cutia* (Ferreiros; adjacent to a degraded forest area) (**A**) and *Sítio Limeirinha* (Nazaré da Mata; adjacent to a monitored forest area) (**B**). The top section represents age groups (indicated by different colors), while the bottom section shows woody medicinal plant species (each represented by blue). Bars connect generations to plant species, illustrating knowledge transmission

We examined how the knowledge of woody medicinal plants is transmitted across age groups through cultural transmission networks. In *Sítio Cutia* (adjacent to a degraded forest area), the cultural transmission network of the learning vector showed that angico was the most cited woody medicinal plant across age groups (274 interactions), followed by aroeira (104), caju roxo, and barbatimão (both 69) (Fig. 4A; Table S1 – Supplementary Material). In the teaching vector, aroeira (82) and caju roxo (64) were the most frequently transmitted species (Fig. 5A; Table S1 – Supplementary Material). Similarly, in *Sítio Limeirinha* (adjacent to a monitored forest area), the learning vector network highlighted aroeira (374), jenipapo (261), juá (255), barbatimão (251), mutamba (138), caju roxo (182), and angico (107) as the most cited species (Fig. 4B; Table S1 – Supplementary Material). The teaching vector network followed a similar pattern, with aroeira (464), jenipapo (334), juá (328), barbatimão (324), jatobá (304), caju roxo (301), mutamba (217), and angico (157) being the most frequently transmitted (Fig. 5B; Table S1 – Supplementary Material).

On the one hand, the degree distribution of the learning vector network at higher levels in *Sítio Cutia* (adjacent to the degraded forest area) follows a truncated power-law distribution (AIC: −19.57; Table S4 – Supplementary Material). This makes it susceptible to the removal of highly connected nodes but reduces the likelihood of disrupting the cultural transmission of LEK. However, the degree distribution of the teaching vector network at higher levels follows a power-law distribution (AIC: −20.59; Table S4 – Supplementary Material), making it highly vulnerable to the removal of key nodes and increasing the risk of disrupting LEK transmission. On the other hand, in *Sítio Limeirinha* (adjacent to the monitored forest area), the learning vector network at higher levels also follows a truncated power-law distribution (AIC: −12.91; Table S4 – Supplementary Material), meaning it is susceptible to node removal but maintains a lower risk of disrupting LEK transmission compared to the network at *Sítio Cutia*. However, the teaching vector network at higher levels in *Sítio Limeirinha* follows an exponential distribution (AIC: 6.18; Table S4 – Supplementary Material), making it highly resistant to node removal and minimizing the probability of disrupting LEK transmission.

## Discussion

Understanding how forest degradation influences the SBS is necessary for assessing how protected areas or the enforcement of specific forest management restrictions could serve as strategies to mitigate forest degradation. In our case study, contrary to our first hypothesis, we found no clear influence of forest degradation on SBS, as both the number of known medicinal woody plant species and perceptions of their availability remained relatively consistent across age groups, regardless of forest degradation status (i.e., degraded vs. monitored forested areas). This challenges the assumption that more degraded landscapes intensify SBS. Although knowledge of current medicinal woody plants does not capture all original environmental conditions, consistent knowledge across age groups indicates that information about historically present species has been maintained, allowing us to infer aspects of past environmental conditions despite degradation. However, in accordance with our second hypothesis, we found that the cultural transmission of LEK has the potential to be more resilient against SBS in areas adjacent to monitored or protected forests than in those adjacent to degraded areas. In these environments, where ecological processes are maintained with less anthropogenic influence, knowledge holders are more likely to maintain and effectively transmit accurate environmental information across different future scenarios of environmental change, reinforcing the continuity of LEK.

Research has examined the interplay between forest degradation, SBS, and the transmission of LEK (e.g., Hanazaki et al. 2013; Fernández-Llamazares et al. 2015). Findings often contrast with our case study, particularly regarding the impact of habitat degradation on knowledge across age groups (e.g., Hanazaki et al. 2013; Fernández-Llamazares et al. 2015). As a major driver of defaunation and deforestation, degradation contributes to age disparities in the recognition of species loss, with younger individuals often perceiving fewer declines in trees, fish, birds, and game vertebrates than older ones (Fernández-Llamazares et al. 2015). In the Tsimane communities of Beni, in the Bolivian Amazon, environmental degradation, combined with climate change, has disrupted traditional ways of life, leading to a decline in younger individuals’ familiarity with cultural practices that shape human-nature interactions (Fernández-Llamazares et al. 2015). Additionally, a review of ethnobotanical studies (Hanazaki et al. 2013) covering various forest resources, including medicinal plants, wild foods, and non-timber products, found that over half of the literature documented SBS through age-related differences in knowledge. Some of these shifts may be attributed to ecological changes, including forest degradation and the reduced availability of traditional resources, ultimately impacting LEK transmission systems (Hanazaki et al. 2013).

Contrary to expectations, our findings revealed no clear influence of forest degradation on SBS. One possible explanation for this pattern is that the persistence of ecological perceptions is not solely dictated by environmental degradation but is also shaped by cultural and social resilience (Jardine 2019). The continued transmission of LEK through learning across age groups, oral traditions, and direct subsistence interactions with the environment may buffer against the expected erosion of baseline perceptions (Hedges et al. 2023; Mota et al. 2023; Soga and Gaston 2024). In many communities, elders serve as key knowledge holders, preserving historical environmental references and ensuring continuity in perceptions of ecological change, regardless of degradation status (Fernández-Llamazares et al. 2015; Viscogliosi et al. 2020). Additionally, the pace of environmental change may be gradual enough that perceptual differences do not emerge sharply between age groups. It is also possible that communities studied have developed adaptive strategies that mitigate the immediate perceptual effects of degradation, maintaining a relatively stable understanding of ecological shifts over time. These strategies may be particularly relevant in the Atlantic Forest region, where habitat loss and fragmentation have historically driven extensive degradation (Vancine et al. 2024), potentially shaping how local populations perceive and respond to environmental changes. In addition, the species mentioned in the interviews are commonly found in small orchards on rural properties (pers. comm.), so even in degraded landscapes, they remain familiar and are still recognized and used as medicinal plants. These factors highlight the need to consider not only ecological conditions but also cultural mechanisms when assessing the influence of degradation on SBS.

However, the contribution of cultural transmission of LEK to resilience against SBS may depend on future ecological conditions. We found that LEK has the potential to be more resilient against SBS in areas adjacent to monitored or protected forests than in those near degraded landscapes. This suggests that the maintenance of conserved ecological dynamics plays a role in sustaining LEK over time, as well-conserved environments may provide more consistent references for knowledge transmission (Cebrián-Piqueras et al. 2020). In contrast, ongoing degradation could reduce the availability of ecological elements essential for learning and passing on traditional knowledge, making LEK more vulnerable in the long term (Jakes 2024). These findings highlight the importance of conservation efforts in ensuring not only the persistence of biodiversity but also the continued transmission of LEK across age groups.

Our findings from this case study suggest that forest degradation does not necessarily drive SBS, as knowledge and perceptions of plant resource availability appeared relatively stable across age groups within the communities examined. This stability may reflect the continued transmission of LEK, which can buffer against the expected erosion of historical environmental baselines. Notably, the resilience of LEK appears to be stronger in areas adjacent to monitored or protected forests, where ecosystem structure and function are more stable, allowing knowledge holders to maintain accurate references for ecological changes. In contrast, ongoing degradation may disrupt key ecological interactions, such as species distributions and resource availability, limiting opportunities for experiential learning and the transfer of knowledge across age groups (Schirpke et al. 2024). Given the limited sample size and the lack of replication across communities, these results should be interpreted. While they provide valuable insights into how degradation might influence LEK and SBS, the study is exploratory and does not allow broad statistical generalization. Nevertheless, the findings emphasize the importance of integrating conservation and knowledge transmission into forest management strategies. Efforts to mitigate SBS should consider not only conserving biodiversity but also maintaining the ecological conditions necessary for LEK to persist. This could include protecting keystone species, ensuring habitat connectivity, and fostering community engagement in conservation initiatives (Soga and Gaston 2024). Additionally, as environmental change accelerates, cultural mechanisms that support LEK transmission, such as participatory monitoring and environmental education programs, may play a critical role in maintaining long-term ecological awareness (Soga and Gaston 2024). By reinforcing the link between social-ecological resilience and LEK, conservation strategies can help sustain both biodiversity and the cultural frameworks that shape human-environment interactions.

## Supplementary Information

Supplementary material

## Data Availability

The data supporting the results can be found on Mendeley Data: 10.17632/528y6vrrvr.1

## References

[CR1] Adla K, Dejan K, Neira D, Dragana S (2022) Degradation of ecosystems and loss of ecosystem services. In: Prata JC, Ribeiro AI, Rocha-Santos T (eds) One health: integrated approach to 21st century challenges to health. Academic Press, Waltham, MA, pp 281–327

[CR2] Ainsworth CH, Pitcher TJ, Rotinsulu C (2008) Evidence of fishery depletions and shifting cognitive baslines in Eastern Indonesia. Biol Conserv 141:848–859. 10.1016/j.biocon.2008.01.006

[CR3] Albert R, Barabási A-L (2002) Statistical mechanics of complex networks. Rev Mod Phys 74:47. 10.1103/RevModPhys.74.47

[CR4] Albuquerque UP, Ramos MA, Lucena RFP, Alencar NL (2014) Methods and techniques used to collect ethnobiological data. In: Albuquerque UP, Cunha LVFC, Lucena RFP, Alves RR (eds) Methods and techniques in ethnobiology and ethnoecology. Springer New York LLC, New York, pp 15–37

[CR5] Alessa L, Kliskey A, Lammers R, Arp C, White D, Hinzman L, Busey R (2008) The Arctic water resource vulnerability index: an integrated assessment tool for community resilience and vulnerability with respect to freshwater. Environ Manag 42:523–541. 10.1007/s00267-008-9152-0 10.1007/s00267-008-9152-0 18560929

[CR6] Anderson MJ (2017) Permutational multivariate analysis of variance (PERMANOVA). Wiley StatsRef: Statistics Reference Online. 10.1002/9781118445112.stat07841

[CR7] Andrade LA, Fabricante JR, Oliveira FX (2009) Invasão biológica por *Prosopis juliflora* (Sw.) DC.: impactos sobre a diversidade e a estrutura do componente arbustivo-arbóreo da caatinga no estado do Rio Grande do Norte, Brasil. Acta Bot Bras 23:935–943. 10.1590/S0102-33062009000400004

[CR8] Araújo IVO, Centeno-Alvarado D, Ramos MA (2024) Access restrictions to forest resources, rather than COVID-19 bans, drive the selection of firewood species for bonfires during *Festas Juninas* in northeastern Brazil. J Ethnobiol Ethnomed 20:41. 10.1186/s13002-024-00677-w 38575934 10.1186/s13002-024-00677-w PMC10996119

[CR9] Associação dos Jovens Agricultores de Portugal (2017) Manual boa práticas para culturas emergentes: a cultura da Romã. Associação dos Jovens Agricultores de Portugal, Lisboa

[CR10] Ávila-Thieme MI, Kusch E, Corcoran D, Castillo SP, Valdovinos FS, Navarrete SA, Marquet PA (2023) NetworkExtinction: an R package to simulate extinction propagation and rewiring potential in ecological networks. Methods Ecol Evol 14:1952–1966. 10.1111/2041-210X.14126

[CR11] Bilney RJ (2014) Poor historical data drive conservation complacency: the case of mammal decline in south-eastern Australian forests. Austral Ecol 39:875–886. 10.1111/aec.12145

[CR12] Burnham KP, Anderson DR (2002) Model selection and multimodel inference: a practical information-theoretic approach. Springer, New York

[CR13] Cebrián-Piqueras MA, Filyushkina A, Johnson DN, Lo VB, López-Rodríguez MD, March H, Oteros-Rozas E, Peppler-Lisbach C, Quintas-Soriano C, Raymond CM, Ruiz-Mallén I, Riper CJ, Zinngrebe Y, Plieninger T (2020) Scientific and local ecological knowledge, shaping perceptions towards protected areas and related ecosystem services. Landsc Ecol 35:2549–2567. 10.1007/s10980-020-01107-4

[CR14] Corcoran D, Ávila-Thieme MI, Valdovinos FS, Navarrete SA, Marquet PA, Kusch E (2023) NetworkExtinction: extinction simulation in ecological networks. R package 1.0.3. Available from: https://cran.r-project.org/web/packages/NetworkExtinction/index.html

[CR15] Davis CC, Choisy P (2024) Medicinal plants meet modern biodiversity science. Curr Biol 34:R158–R173. 10.1016/j.cub.2023.12.038 38412829 10.1016/j.cub.2023.12.038

[CR16] Dias TC, Silveira LF, Francisco MR (2023) Spatiotemporal dynamics reveals forest rejuvenation, fragmentation, and edge effects in an Atlantic Forest hotspot, the Pernambuco Endemism Center, northeastern Brazil. PLoS ONE 18:e0291234. 10.1371/journal.pone.0291234 37682943 10.1371/journal.pone.0291234 PMC10490850

[CR17] Dormann CF, Fruend J, Gruber B, Beckett S, Devoto M, Felix GMF, Iriondo JM, Opsahl T, Pinheiro RBP, Strauss R, Vazquez DP (2024) bipartite: visualising bipartite networks and calculating some (ecological) indices. R. package 2.20. Available from: https://cran.r-project.org/web/packages/bipartite/

[CR18] Dunne JA, Williams RJ, Martinez ND (2002) Network structure and biodiversity loss in food webs: robustness increases with connectance. Ecol Lett 5:558–567. 10.1046/j.1461-0248.2002.00354.x

[CR19] Ellis EC, Goldewijk KK, Siebert S, Lightman D, Ramankutty N (2010) Anthropogenic transformation of the biomes, 1700 to 2000. Glob Ecol Biogeogr 19:589–606. 10.1111/j.1466-8238.2010.00540.x

[CR20] Estrada E (2007) Food webs robustness to biodiversity loss: the roles of connectance, expansibility and degree distribution. J Theor Biol 244:296–307. 10.1016/j.jtbi.2006.08.002 16987531 10.1016/j.jtbi.2006.08.002

[CR21] Fernández-Llamazares A, Díaz-Reviriego I, Luz AC, Cabeza M, Pyhälä A, Reyes-García V (2015) Rapid Ecosystem change challenges the adapative capacity of local environmental knowledge. Glob Environ Chang 31:272–284. 10.1016/j.gloenvcha.2015.02.001 10.1016/j.gloenvcha.2015.02.001 PMC4471143 26097291

[CR22] Freitas WK, Magalhães LMS, Resende AS, Brasil FC, Vivès LR, Pinheiro MAS, Filho PL, Luz RV (2017) Invasion impact of *Artocarpus heterophyllus* Lam. (Moraceae) at the edge of an Atlantic Forest fragment in the municipality of Rio de Janeiro, Brazil. Biosci J. 10.14393/BJ-v33n2-33520

[CR23] Ghazoul J, Burivalova Z, Garcia-Uloa J, King LA (2015) Conceptualizing forest degradation. Trends Ecol Evol 30:622–632. 10.1016/j.tree.2015.08.001 26411619 10.1016/j.tree.2015.08.001

[CR24] Gunn BF, Baudouin L, Olsen KM (2011) Independent origins of cultivated coconut (*Cocos nucifera* L.) in the Old World Tropics. PLoS ONE 6:e21143. 10.1371/journal.pone.0021143 21731660 10.1371/journal.pone.0021143 PMC3120816

[CR25] Hanazaki N, Firme Herbst D, Marques MS, Vandebroek I (2013) Evidence of the shifting baseline syndrome in ethnobotanical research. J Ethnobiol Ethnomed 9:75–86. 10.1186/1746-4269-9-75 24229063 10.1186/1746-4269-9-75 PMC3842669

[CR26] Hansen MC, Potapov PV, Moore R, Hancher, Turubanova SA, Tyukavina A, Thau D, Stehman SV, Goetz SJ, Loveland TR, Kommareddy A, Egorov A, Chini L, Justice CO, Townshend JRG (2013) High-resolution global maps of 21st-century forest cover change. Science 342:850–853. 10.1126/science.1244693 24233722 10.1126/science.1244693

[CR27] Hayhow D, Eaton MA, Stanbury A, Burns F, Kirby W, Bailey N, Beckmann B, Bedford J, Boersch-Supan PH, Coomber F, Dennis EB, Dolman SJ, Dunn E, Hall J, Harrower C, Hatfield JH, Hawley J, Haysom K, Hughes J, Johns DG, Mathews F, McQuatters-Gollop A, Noble DB, Outwaite CL, Pearce-Higgins JW, Pescott OL, Powney GD, Symes N (2019) The state of nature 2019. The State of Nature partnership. National Biodiversity Network Trust, Holborn, London

[CR28] Hedges K, Kipila JO, Carriedo-Ostos R (2023) “There are no trees here”: Understanding perceived intergenerational erosion of traditional medicinal knowledge among Kenyan Purko Maasai in Narok District. J Ethnobiol 40:535–551. 10.2993/0278-0771-40.4.53

[CR29] Herman-Mercer NM, Matkin E, Laituri MJ, Toohey RC, Massey M, Elder K, Schuster PF, Mutter EA (2016) Changing times, changing stories: generational differences in climate change perspectives from four remote indigenous communities in Subarctic Alaska. Ecol Soc 21:28. 10.5751/ES-08463-210328

[CR30] Iniesta-Arandia I, Amo DG, García-Nieto AP, Piñeiro C, Montes C, Martín-López B (2015) Factor influencing local ecological knowledge maintenance in Mediterranean watersheds: Insights for environmental policies. Ambio 44:285–296. 10.1007/s13280-014-0556-1 25286985 10.1007/s13280-014-0556-1 PMC4392019

[CR31] Jakes V (2024) The role of traditional knowledge in sustainable development. Int J Humanit Soc Sci 3:40–55. 10.47941/ijhss.2079

[CR32] Jardine TD (2019) Indigenous knowledge as a remedy for shifting baseline syndrome. Front Ecol Environ 17:13–14. 10.1002/fee.1991

[CR33] Jones L, Turvey ST, Massimino D, Papworth S (2020) Investigating the implications of shifting baseline syndrome on conservation. People Nat 2:1131–1144. 10.1002/pan3.10140

[CR34] Katikiro RE (2014) Perceptions on the shifting baseline among coastal fishers of Tanga, Northeast Tanzania. Ocean Coast Manag 91:23–31. 10.1016/j.ocecoaman.2014.01.009

[CR35] Maciel MIS, Mélo E, Lima V, Souza KA, Silva W (2010) Caracterização físico-química de frutos de genótipos de aceroleira (*Malpighia emarginata* D.C.). Ciênc Tecnol Aliment 30:865–869. 10.1590/S0101-20612010000400005

[CR36] Maria TRBC, Biondi D, Behling A, Sousa NJ (2021) Evaluation of Terminalia catappa street trees: a case study in Itanhaém - São Paulo, Brazil. Urban For Urban Green 66:127373. 10.1016/j.ufug.2021.127373

[CR37] Moloise SD, Matamanda AR, Bhanye J (2023) Traditional ecological knowledge and practices for ecosystem conservation and management: the case of savanna ecosystem services in Limpopo, South Africa. Int J Sustain Dev World Ecol 31:29–42. 10.1080/13504509.2023.2249856

[CR38] Moro MF, Westerkamp C, Martins FR (2013) Naturalization and potential impact of the exotic tree *Azadirachta indica* A.Juss. in Northeastern Brazil. Check List 9:153–157. 10.15560/9.1.153

[CR39] Mossa S, Barthélémy M, Stanley HE, Amaral LAN (2002) Truncation of power law behavior in “scale-free” network models due to information filtering. Phys Rev Lett 88:138701. 10.1103/PhysRevLett.88.138701 11955132 10.1103/PhysRevLett.88.138701

[CR40] Mota MRL, Lauer-Leite ID, Novais JS (2023) Intergenerational transmission of traditional ecological knowledge about medicinal plants in a riverine community of the Brazilian Amazon. Polibotánica 10.18387/polibotanica.56.16

[CR67] Mussi LP (2018) Estudo do fruto Syzygium cumini (Myrtaceae): Efeito da maturação, características físico-químicas, secagem e estabilidade de produtos secos. Thesis, Universidade Estadual do Norte Fluminense Darcy Ribeiro

[CR41] Oksanen J, Simpson GL, Blanchet FG, Kindt R, Legendre P, Minchin PR, O’Hara RB, Solymos P, Stevens MHH, Szoecs E, Wgner H, Barbour M, Bedward M, Bolker B, Borcard D, Carvalho G, Chirico M, Caceres M, Durand S, Evangelista HBA, FitzJohn R, Friendly M, Furneaux B, Hannigan G, Hill MO, Lahti L, McGlinn D, Oullette M-H, Cunha ER, Smith T, Stier A, Braak CJFT, Weedon J, Borman T (2022) vegan. R package 2.6-4. Available from: https://cran.r-project.org/web/packages/vegan/

[CR42] Papworth SK, Rist J, Coad L, Milner-Gulland EJ (2009) Evidence for shifting baseline syndrome in conservation. Conserv Lett 2:93–100. 10.1111/j.1755-263X.2009.00049.x

[CR43] Pauly D (1995) Anecdotes and the shifting baseline syndrome of fisheries. Trends Ecol Evol 10:430. 10.1016/S0169-5347(00)89171-5 21237093 10.1016/s0169-5347(00)89171-5

[CR44] Pilgrim SE, Cullen LC, Smith DJ, Pretty J (2008) Ecological knowledge is lost in wealthier communities and countries. Environ Sci Technol 42:1004–1009. 10.1021/es070837vo 18351064 10.1021/es070837v

[CR45] R Core Team (2022) R: a language and environment for statistical computing. R Foundation for Statistical Computing, Vienna. Available from: https://www.r-project.org/

[CR46] R Core Team (2022) stats. R package 4.3.0. Available from: https://www.cran.r-project.org/web/packages/stats/

[CR47] Ranta P, Blom T, Niemelã J, Joensuu E, Siitonen M (1998) The fragmented Atlantic rain forest of Brazil: size, shape, and distribution of forest fragments. Biodivers Conserv 7:385–403. 10.1023/A:1008885813543

[CR48] Ruifei T, Gavin MC (2016) A classification of threats to traditional ecological knowledge and conservation responses. Conserv Soc 14:57–70. 10.4103/0972-4923.182799

[CR49] Sáenz-Arroyo A, Roberts CM, Torre J, Cariño-Olvera M, Enríquez-Andrade RR (2005) Rapidly shifting environmental baselines among fishers of the Gulf of California. Proc R Soc B 272:1957–1962. 10.1098/rspb.2005.3175 10.1098/rspb.2005.3175 PMC1559885 16191603

[CR50] Schirpke U, Ebner M, Tappeiner U (2024) Effects of climate-related environmental changes on non-material benefits from human-nature interactions: a literature review. Ecosyst Serv 69:101650. 10.1016/j.ecoser.2024.101650

[CR51] Siqueira Filho JA, Santos AMM, Leme EMC, Cabral JS (2007) Atlantic Forest fragments and bromeliads in Pernambuco and Alagoas: Distribution, composition, richness and conservation. In: Siqueira Filho JA, Leme EMC (eds) Fragments of the Atlantic Forest of Northeast Brazil: biodiversity, conservation and the bromeliads. Bromeliad Society International, Austin, TX, pp 101–131

[CR52] Soares FV (2011) Brazil’s Bolsa Família: a review. Econ Polit Wkly 46:55–60.

[CR53] Soga M, Gaston KJ (2018) Shifting baseline syndrome: causes, consequences, and implications. Front Ecol Environ 16:222–230. 10.1002/fee.1794

[CR54] Soga M, Gaston KJ (2016) Extinction of experience: the loss of human-nature interactions. Front Ecol Environ 14:94–101. 10.1002/fee.1225

[CR55] Soga M, Gaston KJ (2021) Towards a unified understanding of human-nature interactions. Nat Sustain 5:374–383. 10.1038/s41893-021-00818-z

[CR56] Soga M, Gaston KJ (2024) Global synthesis indicates widespread occurrence of shifting baseline syndrome. BioScience 74:686–694. 10.1093/biosci/biae068 39444512 10.1093/biosci/biae068 PMC11494512

[CR57] Soga M, Gaston KJ, Kubo T (2018) Cross-generational decline in childhood experiences of neighborhood flowering plants in Japan. Landsc Urban Plan 174:55–62. 10.1016/j.landurbplan.2018.02.009

[CR58] Spennemann DHR (2022) The shifting baseline syndrome and generational amnesia in heritage studies. Heritage 5:2007–2027. 10.3390/heritage5030105

[CR59] Tosa MI, Dziedzic EH, Appel CL, Urbina J, Massey A, Ruprecht J, Eriksson CE, Dolliver JE, Lesmeister DB, Betts MG, Peres CA, Levi T (2021) The rapid rise of next-generation natural history. Front Ecol Evol. 10.3389/fevo.2021.698131

[CR60] Tewksbury JJ, Anderson JG, Bakker JD, Billo TJ, Dunwiddie PW, Groom MJ, Hapmton SE, Herman SG, Levey DJ, Machnicki NJ, Rio CM, Power ME, Rowell K, Salomon AK, Stacey L, Trombulak SC, Wheeler TA (2014) Natural history’s place in science and society. BioScience 64:300–310. 10.1093/biosci/biu032

[CR61] Tilman D, Lehman C (2001) Human-caused environmental change: impacts on plant diversity and evolution. Proc Natl Acad Sci USA 98:5433–5440. 10.1073/pnas.091093198 11344290 10.1073/pnas.091093198 PMC33230

[CR62] Vancine MH, Muylaert RL, Niebuhr BB, Oshima JEF, Tonetti V, Bernardo R, Angelo C, Rosa MR, Grohmann CH, Ribeiro MC (2024) The Atlantic Forest of South America: spatiotemporal dynamics of the vegetation and implications for conservation. Biol Conserv 291:110499. 10.1016/j.biocon.2024.110499

[CR63] Velazco SJE, Bedrij NA, Rojas JL, Keller HA, Ribeiro BR, Marco P (2022) Quantifying the role of protected areas for safeguarding the uses of biodiversity. Biol Conserv 268:109525. 10.1016/j.biocon.2022.109525

[CR64] Viscogliosi C, Asselin H, Basile H, Basile S, Borwick K, Couturier Y, Drolet M-J, Gagnon D, Obradovic N, Torrie J, Zhou D, Levasseur M (2020) Importance of Indigenous elders’ contributions to individual and community wellness: results from a scoping review on social participation and intergenerational solidarity. Can J Public Health 11:667–681. 10.17269/s41997-019-00292-3 10.17269/s41997-019-00292-3 PMC7501322 32109314

[CR65] Warschefsky EJ, von Wettberg EJB (2019) Population genomic analysis of mango (*Mangifera indica*) suggests a complex history of domestication. N Phytol 222:2023–2037. 10.1111/nph.15731 10.1111/nph.15731 30730057

[CR66] Yahia, EM, Salih, NK-E (2011) 22 – Tamarind (*Tamarindus indica* L.). In: Postharvest biology and technology of tropical and subtropical fruits. Woodhead Publishing, pp 442–457. 10.1533/9780857092618.442

