# Supplementary material for: Forest Degradation as a Potential Driver of Shifting Baseline Syndrome in Northeastern Brazil: A Case Study
Source: Environ Manage. 2026 Apr 16;76(5):162. doi: 10.1007/s00267-026-02456-7 (PMC13086699; doi:10.1007/s00267-026-02456-7)
Supplement: Supplementary file 1 — Supplementary material [file 267_2026_2456_MOESM1_ESM.docx]

**SUPPLEMENTARY MATERIAL**

**Forest degradation as a potential driver of shifting baseline syndrome in northeastern Brazil: A case study**

Wyllamys Fernandes da Silva^1^*, Diego Centeno-Alvarado^2^*, Taline Cristina da Silva^1,3^, Marcelo Alves Ramos^1,4^

^1^Programa de Pós-Graduação em Etnobiologia e Conservação da Natureza, Universidade Federal Rural de Pernambuco, Recife, Pernambuco, Brazil

^2^Programa de Pós-Graduação em Biologia Vegetal, Universidade Federal de Pernambuco, Recife, Pernambuco, Brazil

^3^Laboratório de Etnobiologia e Conservação de Ecossistemas, Colegiado de Biologia, Universidade Estadual de Alagoas, Palmeira dos Índios, Alagoas, Brazil

^4^Laboratório de Estudos Etnobiológicos, Universidade de Pernambuco, Campus Mata Norte, Nazaré da Mata, Pernambuco, Brazil

***Willamys F. da Silva and Diego Centeno-Alvarado are equal contributors to this work and designated as co-first authors.**

**Corresponding author:** marcelo.alves@upe.br; Laboratório de Estudos Etnobiológicos, Universidade de Pernambuco, Campus Mata Norte, Nazaré da Mata, Pernambuco, Brazil, 55800-000.

**Email:** wyllamys99@gmail.com (W. F. Silva), centenoalvaradodiego@gmail.com (D. Centeno-Alvarado), taline.cristina@uneal.edu.br (T. C. Silva), marcelo.alves@upe.br (M. A. Ramos).

**ORCID**

Wyllamys Fernandes da Silva: https://orcid.org/0000-0003-2830-2513

Diego Centeno-Alvarado: https://orcid.org/0000-0003-0273-8538

Taline Cristina da Silva: https://orcid.org/0000-0001-8131-0059

Marcelo Alves Ramos: https://orcid.org/0000-0001-5823-4385

**Table S1.** Matrices of the total number of medicinal uses and woody plant species associated with the participants from each age group in the communities of *Sítio Cutia* (Ferreiros; adjacent to a degraded forest area) and *Sítio Limeirinha* (Nazaré da Mata; adjacent to a monitored forest area), state of Pernambuco, Brazil.

| Community | Vector | No. | Family | Species | Common name | Age group | | | | | | |
| --- | --- | --- | --- | --- | --- | --- | --- | --- | --- | --- | --- | --- |
|  |  |  |  |  |  | 18-27 | 28-37 | 38-47 | 48-57 | 58-67 | 68-77 | 87-87 |
| *Sítio Cutia* | Learning | 1 | Anacardiaceae | *Anacardium occidentale* L. | Caju roxo | 1 | 8 | 8 | 17 | 10 | 22 | 3 |
|  |  | 2 | Anacardiaceae | *Astronium urundeuva* (M.Allemão) Engl.) | Aroeira | 5 | 15 | 16 | 22 | 12 | 25 | 9 |
|  |  | 3 | Anacardiaceae | *Mangifera indica* L. | Manga | 0 | 0 | 0 | 1 | 0 | 0 | 0 |
|  |  | 4 | Arecaceae | *Cocos nucifera* L. | Coco | 0 | 6 | 2 | 12 | 0 | 0 | 0 |
|  |  | 5 | Arecaceae | *Syagrus* sp. | Coco catolé | 0 | 0 | 0 | 4 | 0 | 0 | 0 |
|  |  | 6 | Bignoniaceae | *Tabebuia* sp. | Pau d'arco | 0 | 0 | 4 | 0 | 0 | 0 | 4 |
|  |  | 7 | Bignoniaceae | *Tabebuia impetiginosa* (Mart. ex DC.) Standl. | Pau d'arco roxo | 0 | 0 | 0 | 0 | 0 | 2 | 3 |
|  |  | 8 | Bignoniaceae | *Tabebuia serratifolia* (Vahl) G.Nichols. | Pau d'arco amarelo | 0 | 0 | 0 | 2 | 0 | 0 | 0 |
|  |  | 9 | Bombacaceae | *Pseudobombax* sp. | Barriguda | 0 | 0 | 0 | 0 | 0 | 5 | 0 |
|  |  | 10 | Capparaceae | *Crataeva tapia* L. | Trapiá | 0 | 0 | 0 | 0 | 0 | 1 | 0 |
|  |  | 11 | Combretaceae | *Terminalia catappa* L. | Castanhola | 0 | 0 | 1 | 0 | 0 | 0 | 0 |
|  |  | 12 | Cordiaceae | *Cordia trichotoma* (Vell.) Arráb. ex Steud. | Frei Jorge | 0 | 0 | 2 | 0 | 0 | 0 | 0 |
|  |  | 13 | Euphorbiaceae | *Croton blanchetianus* Baill. | Marmeleiro | 0 | 1 | 1 | 5 | 2 | 5 | 2 |
|  |  | 14 | Euphorbiaceae | *Jatropha molissima* (Pohl) Baill. | Pinhão branco | 0 | 0 | 2 | 0 | 0 | 0 | 0 |
|  |  | 15 | Fabaceae | *Anadenanthera colubrina* var. *cebil* (Griseb.) Altschul | Angico | 0 | 4 | 216 | 14 | 9 | 19 | 12 |
|  |  | 16 | Fabaceae | *Bauhinia cheilantha* (Bong.) Steud. | Mororó | 0 | 0 | 0 | 0 | 0 | 6 | 5 |
|  |  | 17 | Fabaceae | *Erythrina velutina* Willd. | Mulungu | 0 | 0 | 0 | 8 | 2 | 0 | 0 |
|  |  | 18 | Fabaceae | *Hymenaea courbaril* L. | Jatobá | 0 | 3 | 3 | 5 | 3 | 15 | 8 |
|  |  | 19 | Fabaceae | *Libidibia ferrea* (Mart. ex Tul.) L.P.Queiroz | Jucá | 1 | 1 | 1 | 2 | 1 | 7 | 5 |
|  |  | 20 | Fabaceae | *Machaerium* sp. | Espinheiro | 0 | 0 | 0 | 4 | 0 | 0 | 4 |
|  |  | 21 | Fabaceae | *Senegalia tenuifolia* (L.) Britton & Rose | Calombi | 0 | 2 | 2 | 0 | 1 | 3 | 1 |
|  |  | 22 | Fabaceae | *Stryphnodendron adstringens* (Mart.) Coville | Barbatimão | 1 | 13 | 16 | 5 | 8 | 20 | 6 |
|  |  | 23 | Lythraceae | *Punica granatum L.* | Romã | 1 | 1 | 1 | 0 | 0 | 0 | 0 |
|  |  | 24 | Malvaceae | *Guazuma ulmifolia* Lam. | Mutamba | 0 | 3 | 4 | 1 | 4 | 0 | 0 |
|  |  | 25 | Moraceae | *Artocarpus heterophyllus* Lam. | Jaca | 0 | 0 | 0 | 1 | 0 | 0 | 0 |
|  |  | 26 | Myrtaceae | *Eucalyptus* sp. | Eucalipto | 0 | 0 | 1 | 0 | 1 | 1 | 0 |
|  |  | 27 | Myrtaceae | *Eugenia uniflora* L. | Pitanga | 0 | 1 | 3 | 7 | 3 | 5 | 1 |
|  |  | 28 | Myrtaceae | *Psidium guajava* L. | Goiaba | 0 | 2 | 4 | 9 | 6 | 4 | 1 |
|  |  | 29 | Myrtaceae | *Syzygium cumini* (L.) Skeels | Azeitona | 0 | 0 | 2 | 0 | 0 | 0 | 0 |
|  |  | 30 | Nyctaginaceae | *Guapira noxia* (Netto) Lundell | João mole | 0 | 0 | 2 | 6 | 4 | 6 | 4 |
|  |  | 31 | Rhamnaceae | *Ziziphus joazeiro* Mart*.* | Juá | 1 | 4 | 5 | 3 | 4 | 13 | 10 |
|  |  | 32 | Rubiaceae | *Genipa americana* L. | Jenipapo | 4 | 4 | 8 | 12 | 5 | 15 | 9 |
|  |  | 33 | Sapotaceae | *Sideroxylon obtusifolium* (Roem. & Schult.) T.D.Penn. | Quixaba | 0 | 0 | 7 | 8 | 4 | 17 | 2 |
|  | Teaching | 34 | Anacardiaceae | *Anacardium occidentale* L. | Caju roxo | 0 | 7 | 8 | 19 | 15 | 15 | 0 |
|  |  | 35 | Anacardiaceae | *Astronium urundeuva* (M.Allemão) Engl.) | Aroeira | 0 | 3 | 16 | 30 | 15 | 14 | 4 |
|  |  | 36 | Anacardiaceae | *Mangifera indica* L. | Manga | 0 | 0 | 0 | 1 | 0 | 0 | 0 |
|  |  | 37 | Arecaceae | *Cocos nucifera* L. | Coco | 0 | 6 | 2 | 9 | 0 | 0 | 0 |
|  |  | 38 | Arecaceae | *Syagrus* sp. | Coco catolé | 0 | 0 | 1 | 4 | 0 | 0 | 0 |
|  |  | 39 | Bignoniaceae | *Tabebuia* sp. | Pau d'arco | 0 | 0 | 0 | 0 | 0 | 0 | 4 |
|  |  | 40 | Bignoniaceae | *Tabebuia impetiginosa* (Mart. ex DC.) Standl. | Pau d'arco roxo | 0 | 0 | 5 | 0 | 0 | 0 | 0 |
|  |  | 41 | Euphorbiaceae | *Croton blanchetianus* Baill. | Marmeleiro | 0 | 0 | 0 | 7 | 0 | 0 | 0 |
|  |  | 42 | Euphorbiaceae | *Jatropha molissima* (Pohl) Baill. | Pinhão branco | 0 | 0 | 2 | 0 | 0 | 0 | 0 |
|  |  | 43 | Fabaceae | *Anadenanthera colubrina* var. *cebil* (Griseb.) Altschul | Angico | 0 | 2 | 9 | 15 | 1 | 11 | 10 |
|  |  | 44 | Fabaceae | *Bauhinia cheilantha* (Bong.) Steud. | Mororó | 0 | 0 | 0 | 0 | 0 | 5 | 5 |
|  |  | 45 | Fabaceae | *Erythrina velutina* Willd. | Mulungu | 0 | 0 | 2 | 5 | 0 | 0 | 1 |
|  |  | 46 | Fabaceae | *Hymenaea courbaril* L. | Jatobá | 0 | 2 | 2 | 6 | 2 | 7 | 8 |
|  |  | 47 | Fabaceae | *Libidibia ferrea* (Mart. ex Tul.) L.P.Queiroz | Jucá | 0 | 0 | 1 | 5 | 1 | 6 | 4 |
|  |  | 48 | Fabaceae | *Machaerium* sp. | Espinheiro | 0 | 0 | 0 | 0 | 0 | 0 | 8 |
|  |  | 49 | Fabaceae | *Machaerium aculeatum* Raddi | Espinho de judeu | 0 | 0 | 1 | 0 | 0 | 0 | 0 |
|  |  | 50 | Fabaceae | *Senegalia tenuifolia* (L.) Britton & Rose | Calombi | 0 | 0 | 0 | 0 | 0 | 1 | 1 |
|  |  | 51 | Fabaceae | *Stryphnodendron adstringens* (Mart.) Coville | Barbatimão | 0 | 6 | 6 | 14 | 6 | 15 | 5 |
|  |  | 52 | Fabaceae | *Tamarindus indica* L. | Tamarindo | 0 | 0 | 1 | 0 | 0 | 0 | 0 |
|  |  | 53 | Lythraceae | *Punica granatum L.* | Romã | 0 | 1 | 0 | 0 | 0 | 0 | 0 |
|  |  | 54 | Malvaceae | *Guazuma ulmifolia* Lam. | Mutamba | 0 | 2 | 0 | 4 | 0 | 0 | 0 |
|  |  | 55 | Moraceae | *Artocarpus heterophyllus* Lam. | Jaqueira | 0 | 0 | 0 | 1 | 0 | 0 | 0 |
|  |  | 56 | Myrtaceae | *Eugenia uniflora* L. | Pitanga | 0 | 0 | 1 | 12 | 2 | 4 | 0 |
|  |  | 57 | Myrtaceae | *Plinia cauliflora* (Mart.) Kausel | Jabuticaba | 0 | 0 | 0 | 3 | 3 | 0 | 0 |
|  |  | 58 | Myrtaceae | *Psidium guajava* L. | Goiaba | 0 | 0 | 0 | 7 | 6 | 4 | 0 |
|  |  | 59 | Myrtaceae | *Psidium guineense* Sw. | Araçá | 0 | 0 | 2 | 0 | 0 | 0 | 0 |
|  |  | 60 | Myrtaceae | *Syzygium cumini* (L.) Skeels | Azeitona | 0 | 0 | 0 | 2 | 0 | 0 | 0 |
|  |  | 61 | Nyctaginaceae | *Guapira noxia* (Netto) Lundell | João mole | 0 | 0 | 0 | 4 | 0 | 3 | 4 |
|  |  | 62 | Rhamnaceae | *Ziziphus joazeiro* Mart*.* | Juá | 0 | 4 | 4 | 19 | 6 | 13 | 10 |
|  |  | 63 | Rubiaceae | *Genipa americana* L. | Jenipapo | 0 | 1 | 7 | 18 | 0 | 9 | 0 |
|  |  | 64 | Sapotaceae | *Sideroxylon obtusifolium* (Roem. & Schult.) T.D.Penn. | Quixaba | 0 | 0 | 4 | 6 | 9 | 9 | 0 |
| *Sítio Limeirinha* | Learning | 65 | Anacardiaceae | *Anacardium occidentale* L. | Caju roxo | 7 | 27 | 52 | 45 | 33 | 18 | 0 |
|  |  | 66 | Anacardiaceae | *Astronium urundeuva* (M.Allemão) Engl.) | Aroeira | 22 | 33 | 74 | 55 | 165 | 19 | 6 |
|  |  | 67 | Anacardiaceae | *Mangifera indica* L. | Manga | 2 | 2 | 0 | 8 | 4 | 0 | 0 |
|  |  | 68 | Anacardiaceae | *Spondias dulcis* Parkinson | Cajarana | 0 | 0 | 0 | 0 | 0 | 0 | 4 |
|  |  | 69 | Anacardiaceae | *Spondias purpurea* L. | Seriguela | 0 | 1 | 0 | 0 | 0 | 0 | 0 |
|  |  | 70 | Arecaceae | *Acrocomia intumescens* Drude | Macaíba | 3 | 6 | 12 | 6 | 18 | 0 | 0 |
|  |  | 71 | Arecaceae | *Cocos nucifera* L. | Coco | 0 | 0 | 0 | 0 | 6 | 0 | 0 |
|  |  | 72 | Arecaceae | *Elaeis guineensis* Jacq. | Coco dendê | 0 | 2 | 0 | 2 | 2 | 0 | 0 |
|  |  | 73 | Arecaceae | *Syagrus* sp. | Coco catolé | 0 | 0 | 0 | 0 | 6 | 0 | 0 |
|  |  | 74 | Bignoniaceae | *Tabebuia* sp. | Pau d'arco | 0 | 0 | 0 | 0 | 0 | 1 | 0 |
|  |  | 75 | Bignoniaceae | *Tabebuia impetiginosa* (Mart. ex DC.) Standl. | Pau d'arco roxo | 0 | 0 | 0 | 2 | 0 | 4 | 0 |
|  |  | 76 | Cordiaceae | *Cordia trichotoma* (Vell.) Arráb. ex Steud. | Frei Jorge | 0 | 0 | 4 | 0 | 0 | 0 | 0 |
|  |  | 77 | Euphorbiaceae | *Jatropha molissima* (Pohl) Baill. | Pinhão branco | 2 | 0 | 0 | 2 | 0 | 0 | 0 |
|  |  | 78 | Fabaceae | *Anadenanthera colubrina* var. *cebil* (Griseb.) Altschul | Angico | 1 | 4 | 10 | 32 | 53 | 1 | 6 |
|  |  | 79 | Fabaceae | *Bauhinia cheilantha* (Bong.) Steud. | Mororó | 0 | 0 | 0 | 0 | 6 | 0 | 0 |
|  |  | 80 | Fabaceae | *Caesalpinia echinata* Lam. | Pau Brasil | 0 | 0 | 0 | 0 | 8 | 0 | 0 |
|  |  | 81 | Fabaceae | *Erythrina velutina* Willd. | Mulungu | 0 | 1 | 0 | 2 | 34 | 0 | 0 |
|  |  | 82 | Fabaceae | *Hymenaea courbaril* L. | Jatobá | 13 | 23 | 79 | 35 | 83 | 8 | 0 |
|  |  | 83 | Fabaceae | *Libidibia ferrea* (Mart. ex Tul.) L.P.Queiroz | Jucá | 0 | 0 | 6 | 2 | 16 | 0 | 4 |
|  |  | 84 | Fabaceae | *Machaerium* sp. | Espinheiro | 0 | 0 | 15 | 7 | 26 | 0 | 0 |
|  |  | 85 | Fabaceae | *Machaerium aculeatum* Raddi | Espinho de judeu | 0 | 0 | 2 | 0 | 0 | 0 | 0 |
|  |  | 86 | Fabaceae | *Senegalia tenuifolia* (L.) Britton & Rose | Calombi | 0 | 0 | 0 | 5 | 2 | 0 | 0 |
|  |  | 87 | Fabaceae | *Stryphnodendron adstringens* (Mart.) Coville | Barbatimão | 13 | 33 | 24 | 52 | 108 | 17 | 4 |
|  |  | 88 | Fabaceae | *Swartzia flaemingii* Raddi | Jacarandá | 0 | 0 | 0 | 1 | 0 | 0 | 0 |
|  |  | 89 | Fabaceae | *Tamarindus indica* L. | Tamarindo | 0 | 0 | 0 | 4 | 2 | 1 | 0 |
|  |  | 90 | Lythraceae | *Punica granatum L.* | Romã | 1 | 12 | 0 | 4 | 40 | 0 | 0 |
|  |  | 91 | Malvaceae | *Guazuma ulmifolia* Lam. | Mutamba | 4 | 17 | 32 | 30 | 38 | 13 | 4 |
|  |  | 92 | Meliaceae | *Azadirachta indica* A.Juss. | Nim | 0 | 3 | 0 | 0 | 0 | 0 | 0 |
|  |  | 93 | Myrtaceae | *Eucalyptus* sp. | Eucalipto | 0 | 4 | 12 | 2 | 0 | 0 | 0 |
|  |  | 94 | Myrtaceae | *Eugenia uniflora* L. | Pitanga | 0 | 0 | 5 | 0 | 18 | 0 | 0 |
|  |  | 95 | Myrtaceae | *Psidium guajava* L. | Goiaba | 1 | 1 | 21 | 4 | 22 | 0 | 1 |
|  |  | 96 | Myrtaceae | *Syzygium cumini* (L.) Skeels | Azeitona | 0 | 0 | 0 | 0 | 2 | 0 | 0 |
|  |  | 97 | Sapindaceae | *Cupania impressinervia* Acev.-Rodr. | Cabatam | 0 | 0 | 0 | 2 | 0 | 0 | 0 |
|  |  | 98 | Rhamnaceae | *Ziziphus joazeiro* Mart*.* | Juá | 5 | 26 | 70 | 48 | 90 | 16 | 0 |
|  |  | 99 | Rubiaceae | *Genipa americana* L. | Jenipapo | 7 | 27 | 73 | 37 | 89 | 21 | 7 |
|  |  | 100 | Sapotaceae | *Sideroxylon obtusifolium* (Roem. & Schult.) T.D.Penn. | Quixaba | 0 | 0 | 0 | 0 | 16 | 0 | 0 |
|  |  | 101 | Urticaceae | *Cecropia* sp. | Embaúba | 0 | 0 | 6 | 8 | 0 | 0 | 0 |
|  | Teaching | 102 | Anacardiaceae | *Anacardium occidentale* L. | Caju roxo | 6 | 25 | 75 | 69 | 92 | 28 | 6 |
|  |  | 103 | Anacardiaceae | *Astronium urundeuva* (M.Allemão) Engl.) | Aroeira | 11 | 26 | 100 | 72 | 192 | 63 | 0 |
|  |  | 104 | Anacardiaceae | *Mangifera indica* L. | Manga | 2 | 0 | 3 | 9 | 4 | 6 | 0 |
|  |  | 105 | Anacardiaceae | *Spondias dulcis* Parkinson | Cajarana | 0 | 0 | 0 | 0 | 0 | 0 | 4 |
|  |  | 106 | Anacardiaceae | *Spondias purpurea* L. | Seriguela | 0 | 1 | 1 | 0 | 0 | 0 | 0 |
|  |  | 107 | Arecaceae | *Acrocomia intumescens* Drude | Macaíba | 3 | 3 | 20 | 6 | 22 | 2 | 3 |
|  |  | 108 | Arecaceae | *Cocos nucifera* L. | Coco | 0 | 0 | 0 | 0 | 8 | 0 | 0 |
|  |  | 109 | Arecaceae | *Elaeis guineensis* Jacq. | Coco dendê | 0 | 2 | 6 | 2 | 6 | 0 | 0 |
|  |  | 110 | Arecaceae | *Syagrus* sp. | Coco catolé | 0 | 0 | 0 | 3 | 4 | 2 | 0 |
|  |  | 111 | Euphorbiaceae | *Jatropha molíssima* (Pohl) Baill. | Pinhão branco | 2 | 0 | 0 | 10 | 2 | 1 | 0 |
|  |  | 112 | Bignoniaceae | *Tabebuia* sp. | Pau d'arco | 0 | 0 | 0 | 6 | 2 | 1 | 0 |
|  |  | 113 | Bignoniaceae | *Tabebuia impetiginosa* (Mart. ex DC.) Standl. | Pau d'arco roxo | 0 | 0 | 0 | 10 | 11 | 7 | 0 |
|  |  | 114 | Bignoniaceae | *Tabebuia serratifolia* (Vahl) G.Nichols. | Pau d'arco amarelo | 0 | 0 | 0 | 0 | 8 | 0 | 0 |
|  |  | 115 | Capparaceae | *Crataeva tapia* L. | Trapiá | 0 | 0 | 0 | 1 | 0 | 0 | 0 |
|  |  | 116 | Combretaceae | *Thiloa glaucocarpa* (Mart.) Eichler | Sipaúba | 0 | 0 | 4 | 4 | 14 | 2 | 0 |
|  |  | 117 | Cordiaceae | *Cordia trichotoma* (Vell.) Arráb. ex Steud. | Frei Jorge | 0 | 0 | 4 | 0 | 0 | 0 | 0 |
|  |  | 118 | Fabaceae | *Anadenanthera colubrina* var. *cebil* (Griseb.) Altschul | Angico | 0 | 7 | 4 | 46 | 66 | 26 | 8 |
|  |  | 119 | Fabaceae | *Bauhinia cheilantha* (Bong.) Steud. | Mororó | 0 | 0 | 0 | 0 | 6 | 0 | 0 |
|  |  | 120 | Fabaceae | *Bowdichia virgilioides* Kunth | Sucupira | 0 | 0 | 0 | 0 | 3 | 6 | 0 |
|  |  | 121 | Fabaceae | *Caesalpinia echinata* Lam. | Pau Brasil | 0 | 0 | 0 | 0 | 8 | 0 | 0 |
|  |  | 122 | Fabaceae | *Erythrina velutina* Willd. | Mulungu | 0 | 0 | 3 | 10 | 49 | 19 | 0 |
|  |  | 123 | Fabaceae | *Hymenaea courbaril* L. | Jatobá | 11 | 13 | 93 | 49 | 112 | 25 | 1 |
|  |  | 124 | Fabaceae | *Inga* *vera* Willd. | Ingá | 0 | 0 | 12 | 0 | 0 | 0 | 0 |
|  |  | 125 | Fabaceae | *Libidibia ferrea* (Mart. ex Tul.) L.P.Queiroz | Jucá | 0 | 0 | 9 | 2 | 24 | 4 | 4 |
|  |  | 126 | Fabaceae | *Machaerium* sp. | Espinheiro | 0 | 0 | 10 | 8 | 38 | 7 | 0 |
|  |  | 127 | Fabaceae | *Piptadenia retusa* (Jacq.) P.G.Ribeiro, Seigler & Ebinger | Jurema | 0 | 0 | 3 | 0 | 0 | 0 | 0 |
|  |  | 128 | Fabaceae | *Senegalia tenuifolia* (L.) Britton & Rose | Calombi | 0 | 0 | 2 | 4 | 2 | 0 | 0 |
|  |  | 129 | Fabaceae | *Stryphnodendron adstringens* (Mart.) Coville | Barbatimão | 4 | 24 | 35 | 78 | 141 | 31 | 11 |
|  |  | 130 | Fabaceae | *Tamarindus indica* L. | Tamarindo | 0 | 0 | 0 | 2 | 2 | 0 | 3 |
|  |  | 131 | Lythraceae | *Punica granatum L.* | Romã | 0 | 11 | 0 | 4 | 40 | 0 | 0 |
|  |  | 132 | Malpighiaceae | *Malpighia emarginata* DC. | Acerola | 0 | 0 | 0 | 0 | 0 | 2 | 0 |
|  |  | 133 | Malvaceae | *Guazuma ulmifolia* Lam. | Mutamba | 1 | 15 | 34 | 60 | 57 | 43 | 7 |
|  |  | 134 | Meliaceae | *Azadirachta indica* A.Juss. | Nim | 0 | 3 | 0 | 0 | 0 | 0 | 0 |
|  |  | 135 | Myrtaceae | *Eucalyptus* sp. | Eucalipto | 0 | 1 | 12 | 2 | 8 | 0 | 0 |
|  |  | 136 | Myrtaceae | *Eugenia uniflora* L. | Pitanga | 0 | 0 | 10 | 0 | 24 | 0 | 0 |
|  |  | 137 | Myrtaceae | *Psidium guajava* L. | Goiaba | 0 | 1 | 25 | 8 | 27 | 19 | 0 |
|  |  | 138 | Myrtaceae | *Syzygium cumini* (L.) Skeels | Azeitona | 0 | 2 | 0 | 0 | 6 | 0 | 0 |
|  |  | 139 | Nyctaginaceae | *Guapira noxia* (Netto) Lundell | João mole | 0 | 0 | 0 | 0 | 2 | 2 | 0 |
|  |  | 140 | Rhamnaceae | *Ziziphus joazeiro* Mart*.* | Juá | 0 | 18 | 74 | 63 | 115 | 51 | 7 |
|  |  | 141 | Rubiaceae | *Genipa americana* L. | Jenipapo | 12 | 20 | 79 | 50 | 106 | 57 | 10 |
|  |  | 142 | Sapindaceae | *Cupania impressinervia* Acev.-Rodr. | Cabatam | 0 | 0 | 0 | 0 | 0 | 2 | 0 |
|  |  | 143 | Sapotaceae | *Sideroxylon obtusifolium* (Roem. & Schult.) T.D.Penn. | Quixaba | 0 | 0 | 0 | 0 | 16 | 0 | 0 |
|  |  | 144 | Urticaceae | *Cecropia* sp. | Embaúba | 0 | 0 | 6 | 9 | 0 | 6 | 0 |

**Table S2.** Dunn’s multiple comparisons test (*P*-values) for the richness of woody medicinal plants based on local ecological knowledge (LEK) across age groups in the communities of *Sítio Cutia* (Ferreiros; adjacent to a degraded forest area) and *Sítio Limeirinha* (Nazaré da Mata; adjacent to a monitored forest area), state of Pernambuco, Brazil.

| *Sítio Cutia* | Age group | 18-27 | 28-37 | 38-47 | 48-57 | 58-67 | 68-77 | 78-87 |
| --- | --- | --- | --- | --- | --- | --- | --- | --- |
|  | 18-27 | - | 1 | 1 | 0.08 | 0.75 | 0.07 | 0.10 |
|  | 28-37 | 1 | - | 1 | **0.03** | 1 | **0.04** | 0.12 |
|  | 38-47 | 1 | 1 | - | 0.28 | 1 | 0.28 | 0.52 |
|  | 48-57 | 0.08 | **0.03** | 0.28 | - | 1 | 1 | 1 |
|  | 58-67 | 0.75 | 1 | 1 | 1 | - | 1 | 1 |
|  | 68-77 | 0.07 | **0.04** | 0.28 | 1 | 1 | - | 1 |
|  | 78-87 | 0.10 | 0.12 | 0.52 | 1 | 1 | 1 | - |
| *Sítio Limeirinha* | Age group | 18-27 | 28-37 | 38-47 | 48-57 | 58-67 | 68-77 | 78-87 |
|  | 18-27 | - | 1 | **0.01** | **0.02** | **0.001** | **0.02** | 1 |
|  | 28-37 | 1 | - | 0.46 | 0.52 | **0.001** | 0.43 | 1 |
|  | 38-47 | **0.01** | 0.46 | - | 1 | 0.42 | 1 | 1 |
|  | 48-57 | **0.02** | 0.52 | 1 | - | 0.30 | 1 | 1 |
|  | 58-67 | **0.001** | **0.001** | 0.42 | 0.30 | - | 0.58 | 1 |
|  | 68-77 | **0.02** | 0.43 | 1 | 1 | 0.58 | - | 1 |
|  | 78-87 | 1 | 1 | 1 | 1 | 1 | 1 | - |

**Table S3.** Results of the PERMANOVA analysis on the differences in the perception of the availability of woody medicinal plants across age groups in the communities of *Sítio Cutia* (Ferreiros; adjacent to a degraded forest area) and Sítio Limeirinha (Nazaré da Mata; adjacent to a monitored forest area), state of Pernambuco, Brazil. DF: degrees of freedom; SS: sum of squares; significant values are in bold.

| Community | Variables | DF | SS | R^2^ | *F*-statistic | *p* |
| --- | --- | --- | --- | --- | --- | --- |
| *Sítio Cutia* | Age group | 6 | 24.09 | 0.16 | 1.14 | 0.34 |
|  | Residual | 78 | 274.77 | 0.83 |  |  |
| *Sítio Limeirinha* | Age group | 6 | 4.25 | 0.05 | 0.58 | 0.76 |
|  | Residuals | 114 | 139.98 | 0.95 |  |  |

**Table S4.** Degree distribution of cultural transmission networks for learning and teaching vectors in the communities of *Sítio Cutia* (Ferreiros; adjacent to a degraded forest area) and *Sítio Limeirinha* (Nazaré da Mata; adjacent to a monitored forest area), state of Pernambuco, Brazil.

| Community | Vector | Model | *R^2^* | AIC |
| --- | --- | --- | --- | --- |
| *Sítio Cutia* | Learning | Exponential | 0.85 | -0.61 |
|  |  | Power law | 0.78 | 1.71 |
|  |  | **Truncated power law** | **0.99** | **-19.57** |
|  | Teaching | Exponential | 0.99 | -18.18 |
|  |  | **Power law** | **0.99** | **-20.59** |
|  |  | Truncated power law | 1 | -19.58 |
| *Sítio Limeirinha* | Learning | Exponential | 0.98 | -10.42 |
|  |  | Power law | 0.94 | -4.53 |
|  |  | **Truncated power law** | **0.99** | **-12.91** |
|  | Teaching | **Exponential** | **0.96** | **-6.28** |
|  |  | Power law | 0.93 | -3.29 |
|  |  | Truncated power law | 0.97 | -5.53 |
